# Cancer cell population growth kinetics at low densities deviate from the exponential growth model and suggest an Allee effect

**DOI:** 10.1371/journal.pbio.3000399

**Published:** 2019-08-05

**Authors:** Kaitlyn E. Johnson, Grant Howard, William Mo, Michael K. Strasser, Ernesto A. B. F. Lima, Sui Huang, Amy Brock

**Affiliations:** 1 Department of Biomedical Engineering, The University of Texas at Austin, Austin, Texas, United States of America; 2 Institute for Systems Biology, Seattle, Washington, United States of America; 3 Institute for Computation Engineering and Sciences, The University of Texas at Austin, Austin, Texas, United States of America; 4 Department of Oncology, Livestrong Cancer Institute, Dell Medical School, The University of Texas at Austin, Austin, Texas, United States of America; The Pennsylvania State University, UNITED STATES

## Abstract

Most models of cancer cell population expansion assume exponential growth kinetics at low cell densities, with deviations to account for observed slowing of growth rate only at higher densities due to limited resources such as space and nutrients. However, recent preclinical and clinical observations of tumor initiation or recurrence indicate the presence of tumor growth kinetics in which growth rates scale positively with cell numbers. These observations are analogous to the cooperative behavior of species in an ecosystem described by the ecological principle of the Allee effect. In preclinical and clinical models, however, tumor growth data are limited by the lower limit of detection (i.e., a measurable lesion) and confounding variables, such as tumor microenvironment, and immune responses may cause and mask deviations from exponential growth models. In this work, we present alternative growth models to investigate the presence of an Allee effect in cancer cells seeded at low cell densities in a controlled in vitro setting. We propose a stochastic modeling framework to disentangle expected deviations due to small population size stochastic effects from cooperative growth and use the moment approach for stochastic parameter estimation to calibrate the observed growth trajectories. We validate the framework on simulated data and apply this approach to longitudinal cell proliferation data of BT-474 luminal B breast cancer cells. We find that cell population growth kinetics are best described by a model structure that considers the Allee effect, in that the birth rate of tumor cells increases with cell number in the regime of small population size. This indicates a potentially critical role of cooperative behavior among tumor cells at low cell densities with relevance to early stage growth patterns of emerging and relapsed tumors.

## Abstract 2

This study applied principles that describe the growth dynamics of species within an ecosystem in a novel attempt to understand the growth of tumors. At low cell densities, cooperative interactions among cancer cells may influence growth in a manner reminiscent of the ecological “Allee effect,” in contrast to conventional logistic growth models.

## Introduction

The classical formulation of tumor growth models often begins with the assumption that early stage tumor growth dynamics are driven by cell-autonomous proliferation, manifested as an exponential increase in cell number. The exponential growth model describes a growth rate that is proportional to the number of cells present and is often captured by a single growth rate constant at this stage. However, current imaging technologies have a lower limit of detection of about 1 million cells on a typical CT scan [1], and thus measurements of the growth dynamics of very small tumor cell populations are not typically captured in the clinical setting [1]. Recent findings in preclinical mouse models [2] and from clinical outcomes following tumor resection [3] reveal that tumor growth at low tumor cell densities does not match the expectation of exponential growth. In addition, observations of in vitro cell growth have long recognized that very low seeding density may have a detrimental effect on population fitness. These findings give rise to an intriguing possibility: does tumor cell growth deviate from the model of exponential growth at low tumor cell densities? In this study, we ask whether early stage tumor growth kinetics exhibits a behavior analogous to a principle in ecology known as the Allee effect, in which the fitness of a population, measured by the per capita growth rate, scales with population size at low population sizes. In ecology, the Allee effect arises due to cooperative growth, such as cooperative predation, feeding, and mating systems [4]. In tumors, there exists an abundance of evidence for subclonal interactions among cells, e.g., with specific subpopulations releasing signaling molecules critical to the growth of other subsets of cells [5–9].Thus, it is quite intuitive that cancer cell growth may exhibit cooperative interactions analogous to the cooperation among species in an ecosystem.

The ability to describe and predict tumor growth is essential to developing strategies to eradicate cancer cell populations [10, 11]. Understanding tumor growth kinetics at low cell numbers is of clinical importance because they govern tumor initiation, treatment response, and recurrence. In ecology, the Allee effect has informed strategies for the control of invasive species [12] and has been used to predict how an introduced species might take hold in a new environment [4]. Applying ecological principles to control tumor growth is a growing interest [13–24]. A better understanding of the factors that govern tumor cell growth at early stages could help to improve predictions of initial growth, relapse, and metastasis, as well as guide therapeutic strategies borrowed from ecological principles to control tumor progression.

Although exponential growth is a common initial assumption used to develop more complex models of tumor progression, few models strictly interested in characterizing tumor growth prescribe a fixed birth and death rate over time and population size. Many modifications have been made to account for a changing growth rate as the population grows. The widely used Gompertzian model is a phenomenological model that introduces a growth rate that decays exponentially with time [25–27]. Similarly, the logistic growth model exhibits a modification that introduces a population size dependency, slowing the growth rate as the population size approaches carrying capacity [25, 26]. This can be explained mechanistically as a result of competition over finite space and nutrients. Further refinements to these models have used a statistical mechanics framework to explicitly introduce a correlation function, reducing the number of accessible growth states of individual cells as the population size increases, leading to slowing of growth [17]. Other mechanistic models have used a replicator system of equations for competing species, with population-dependent fitness based on a payoff matrix [28]. Stochastic models of tumor growth have also been used to describe tumor growth based on a Moran birth–death process, a stochastic model that describes how heterogeneity increases over time due to molecular mutations in individual cells [29]. This stochastic modeling framework leads to a population-dependent fitness landscape that exhibits nonconstant tumor growth rates; specifically, tumor growth rates that slow in the later stages of development [29]. Although all of these models take into account decreasing growth rates as the population grows [17, 25–27, 29–31], none explicitly investigate the opposing effect of an increase in growth rate with population size at low population densities.

Recent evidence of deviations from exponential growth at early stages of tumor growth have been observed in glioblastoma, in which patient brain tumors were resected and monitored over time for relapse [3]. These studies of relapsed tumor growth revealed that the observed growth rate at the clinically detectable stages of tumor growth failed to match models of simple logistic growth and instead were better described by a weak Allee effect model. In the weak version of the Allee effect, populations grow at a much slower rate at very low tumor cell numbers but continue to grow for any initial population size. By contrast, a strong Allee effect describes a population that becomes extinct below a threshold initial population size. In ecology, both strong and weak Allee effects are observed [4]. Although the observation of a weak Allee effect in glioblastoma recurrence is certainly provocative, it is limited by the fact that the earliest stages of tumor growth from low cell densities cannot be easily detected in vivo and thus the critical measurements at the relevant regime cannot be captured with current imaging technologies. Numerous studies have investigated the manifestation of the Allee effect in ecology [32–35] and a few have posed theoretical implications and possible mechanisms of cooperative kinetics of the Allee effect in cancer growth [36–40]. However, none have performed an in-depth quantitative analysis of cancer cell proliferation kinetics captured in the low cell density regime. The explicit investigation of the Allee effect in describing tumor growth dynamics at low population sizes is the main contribution of this paper.

In this study, we investigate the behavior of various structurally distinct models of tumor growth representing alternative hypotheses of growth dynamics that consider the Allee effect. We present a framework for the analysis of cancer cell growth at low cell densities in a controlled in vitro setting in which cells are subject to optimal growth conditions with sufficient nutrients and space. Monitoring growth in vitro allows for studying the effects of cell number on growth in the absence of confounding factors, such as the immune system interactions and tissue microenvironmental factors, in order to test explicitly the dependence of growth dynamics on cell density. We take advantage of recent technological advances that allow for the seeding of a precise small initial cell number and the ability to measure cell number at single-cell resolution and at high-temporal resolution. This enables capturing of accurate growth kinetics in the low cell-density regime in which the Allee effect is most relevant and cannot be studied in vivo. Because we focus our examination in the low cell-density regime exclusively (<200 cells in a 1 mm^3^ well), our modeling analysis excludes additional terms that describe the slowing of cancer cell population growth at higher densities in which competition for limited resources and space becomes relevant. We examine the average behavior of 3 models of increasing complexity: the exponential growth model, a strong Allee model, and an extended Allee model that can be either strong or weak.

At the small population size of interest in this study, the inherent stochasticity of the birth–death problem leads to a nonzero probability of extinction, even for a model of constant, net-positive birth rate minus death rate. This phenomena, in which the average population behavior appears to have a reduced growth rate because some trajectories become extinct and have a growth rate of zero, is known in ecology as demographic stochasticity [41]. In order to disentangle these stochastic effects of small population sizes that decrease observed average growth rate from true cooperative effects, we develop 7 stochastic models whose average behavior follows one of the deterministic models. In this framework, each stochastic model represents a different hypothesis of the mechanism underlying the growth kinetics. For each stochastic model, we perform a parameter estimation using the method of moments [42] and use model selection to identify the model most likely model to describe the growth data [42]. This is performed for both a simulated data set and the in vitro BT-474 breast cancer cell line data to test the hypotheses that our framework reveals an alternative tumor growth model that incorporates an Allee effect.

## Results

### The deterministic strong and weak Allee effect models

This work studies stochastic growth models because of the inherent stochasticity of cell growth processes in the regime of small cell numbers that is the focus of our work. However, the structure of the functional forms of the kinetic equations describing the cell-number changes can be understood within a deterministic framework, thus providing a link to the historical and most widely implemented tumor growth models. The deterministic models involving the Allee effect are chosen because they have previously shown to be useful for applied ecologists working in regimes in which the Allee effect is relevant [4].

At the core of our modeling effort are the following 3 deterministic phenomenological models of increasing complexity that describe cell population growth kinetics. The first model represents the null model of tumor growth [26] where the growth rate ($\frac{\partial N}{\partial t}$) is proportional to the number of cells present, *N*, and a single growth rate constant, *g*, resulting in the classical exponential growth model:

$$\frac{\partial N(t)}{\partial t}=gN(t)$$ (1)

$$N(t)=N_{0}e^{gt}$$ (2)

**Fig 1:**
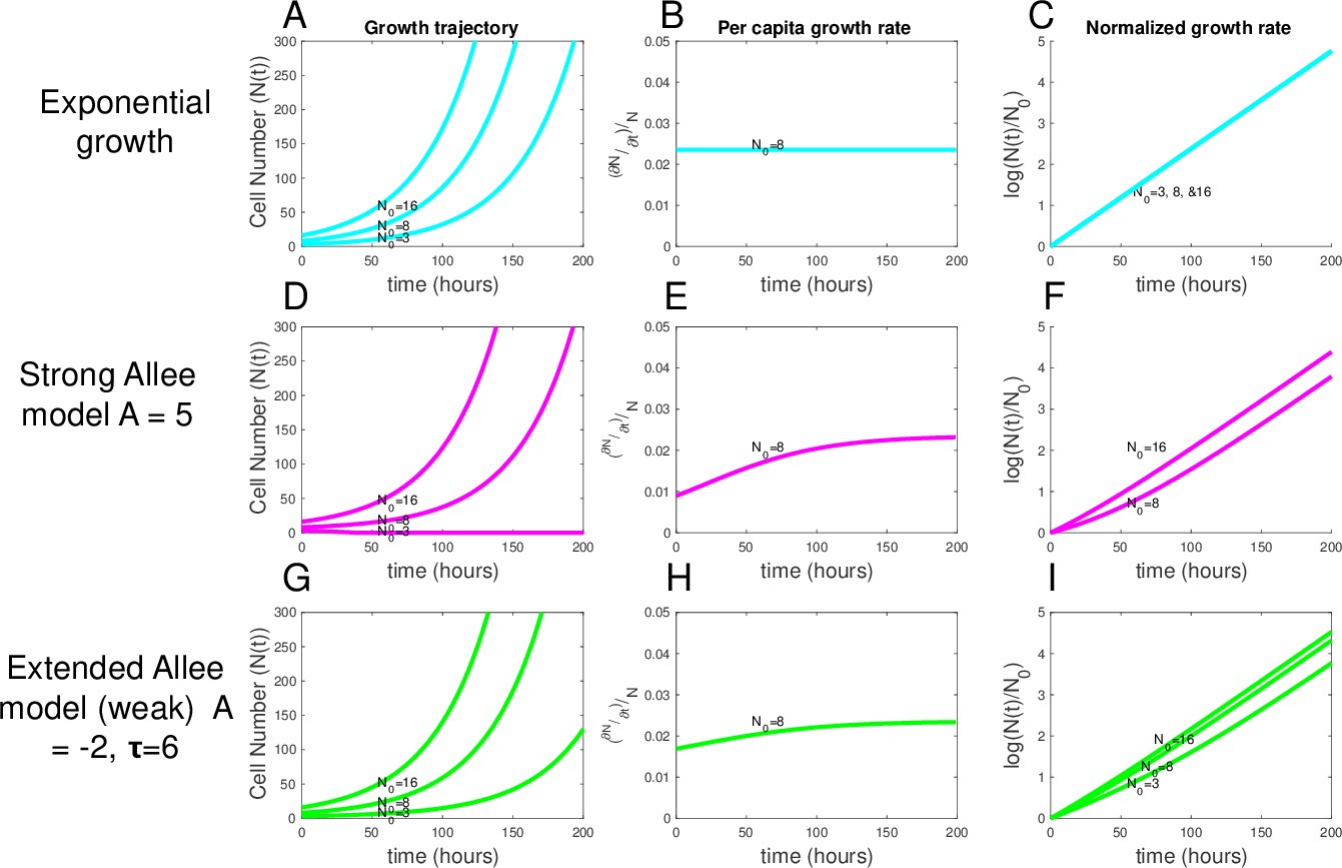
Average behavior of exponential, strong, and weak Allee models for different initial conditions. (A, D, and G) Deterministic growth curves of the exponential growth model (blue), the strong Allee model (pink), and the weak Allee model (green), respectively, shown for N_0_ = 3, 8, and 16 for all models. (B, E, and H) Per capita growth rates demonstrate that growth rate increases in time with cell number for both Allee models. (C, F, and I) For normalized cell numbers, a clear difference is observed in the slopes depending on the initial cell number for both Allee models. Data and code used to generate this figure can be found at https://github.com/brocklab/Johnson-AlleeGrowthModel.git.

This model describes *N* cells that exhibit cell autonomous proliferation (Fig 1A) and a constant per capita growth rate ($\frac{\frac{\partial N}{\partial t}}{N}$) given by the growth rate constant *g* over time and cell number (Fig 1B) for initial cell numbers *N*(*t* = 0) = 3, 8, and 16 cells displayed in (Fig 1A, 1B and 1C). In the remainder of the manuscript we denote the initial cell number, *N*(*t* = 0) by *N*_0_. The normalized growth rate ($log(\frac{N(t)}{N_{0}})$) is constant for each initial condition, with all growth curves falling on a line of equal slope (Fig 1C). Eqs 1 and 2 represent the well-known exponential growth model and the simplest of the tumor growth models analyzed.

Most departures from the exponential growth model of cancer cells (Eqs 1 and 2) describe cancer cell growth in which the growth rate is proportional to the number of cells present but with modifications that account for slowing of growth over time and/or with increasing population size. For example, in the classical formulation of the logistic growth model [26], the growth rate is characterized by a growth rate constant *g* modulated by an additional term to describe the slowing of growth rate as the population approaches carrying capacity (*K*):

$$\frac{\partial N(t)}{\partial t}=g(1-\frac{N(t)}{K})N(t)$$ (3)

The logistic growth model describes cells in 2 regimes: when *N <<K* the $\frac{N}{K}$ term is negligible and the cells essentially exhibit exponential growth, and when *N* is near *K*, the net growth rate ($\frac{\partial N}{\partial t}$) slows towards zero as *N* approaches *K* and the $1-\frac{N}{K}$ term approaches zero.

We present the logistic growth model (Eq 3) to demonstrate that the second model equation (Eq 4), the strong Allee model, is analogous to the logistic growth model, except that the dependency on *N* in this model occurs in the opposite regime—introducing an Allee effect term of $1-\frac{A}{N}$ that lowers the observed growth rate at small *N* near the Allee threshold *A*:

$$\frac{\partial N(t)}{\partial t}=g(1-\frac{A}{N(t)})N(t)$$ (4)

This model describes *N* cells whose net growth rate exists in 2 distinct regimes: when *N* is less than the Allee threshold (*A*), the Allee effect term $1-\frac{A}{N}$ in Eq 4 becomes negative and the net growth rate ($\frac{\partial N}{\partial t}$) becomes negative, predicting the population will ultimately go extinct (Fig 1D; N_0_ = 3). When *N*(*t*) is near *A* but larger than *A*, the net growth rate is slowed by a factor of $1-\frac{A}{N}$ (Eq 4) but remains positive, resulting in a growth rate that scales with cell number, as can be seen for the per capita growth rate over time for *N*_0_ = 8 (Fig 1E). When *N*(*t*) is much larger than *A*, the Allee effect term ($1-\frac{A}{N}$) becomes negligible and the cell population begins to behave like in the exponential growth model (Fig 1D and 1E). This behavior in which a population is predicted to go extinct below a critical threshold (here A) describes a strong Allee effect. The expected scaling of the normalized growth rate ($log(\frac{N(t)}{N_{0}})$) demonstrates the expected differences in net growth rate based on initial seeding number (*N*_0_) for a strong Allee model (Fig 1F). As expected, only initial conditions greater than the Allee threshold of *A* = 5 (corresponding to N_0_ = 8 and 16) result in a net positive growth rate. This model is able to explain the threshold-like behavior observed in preclinical studies of engrafted tumors in mouse [2], where, below a threshold number of inoculated cells, tumors never form. To account for weak Allee effect behavior, in which the growth rate is always greater than zero for any N_0_, we introduce the third deterministic model, the extended Allee model:

$$\frac{\partial N(t)}{\partial t}=g(1-\frac{A+\tau }{N(t)+\tau })N(t)$$ (5)

This model is similar to the strong Allee model (Eq 4) but introduces an additional parameter τ that allows the model to exhibit either a strong Allee effect when *A* is positive, or a weak Allee effect when *τ* > |*A*| and *A* < 0. When weak Allee conditions hold, at low *N* the ($\frac{A+\tau }{N+\tau }$) term always remains less than 1 but greater than zero, keeping the net growth rate positive but resulting in a growth rate that approaches zero as *N* decreases. Fig 1G, 1H and 1I display the behavior of the extended Allee model with parameters that produce a weak Allee effect. The extended Allee model explains potential weak Allee effects, such as those observed in glioblastoma resection [3]. See S1 Table for a complete description of each of the 3 deterministic models, their parameters, and their behaviors.

### Extension to stochastic growth models

Given that the growth kinetics are measured here in very small cell populations, where the expected variability of individual cell behavior with respect to division “birth” and “death” events (which jointly determine net growth rate $\frac{\partial N}{\partial t}$) is high, this scenario can give rise to apparent growth kinetics that deviate significantly from the average population behavior. In order to detect slowing of growth that is not due to stochastic small population effects that result in reduced average observed growth, a stochastic modeling framework was implemented to test the relevance of the Allee effect models presented above. Stochastic models are often derived from microscopic models that describes density-dependent birth and death rates. However, in this approach, we chose the expressions for our stochastic models in order to recapitulate a first-order moment (mean) that matched the corresponding deterministic ordinary differential equation (ODE) of the Allee effect. Birth and death rates were thus chosen not directly from a first-principles derivation of a microscopic model but based on reasonable hypotheses consistent with the Allee effect model behavior.

**Fig 2:**
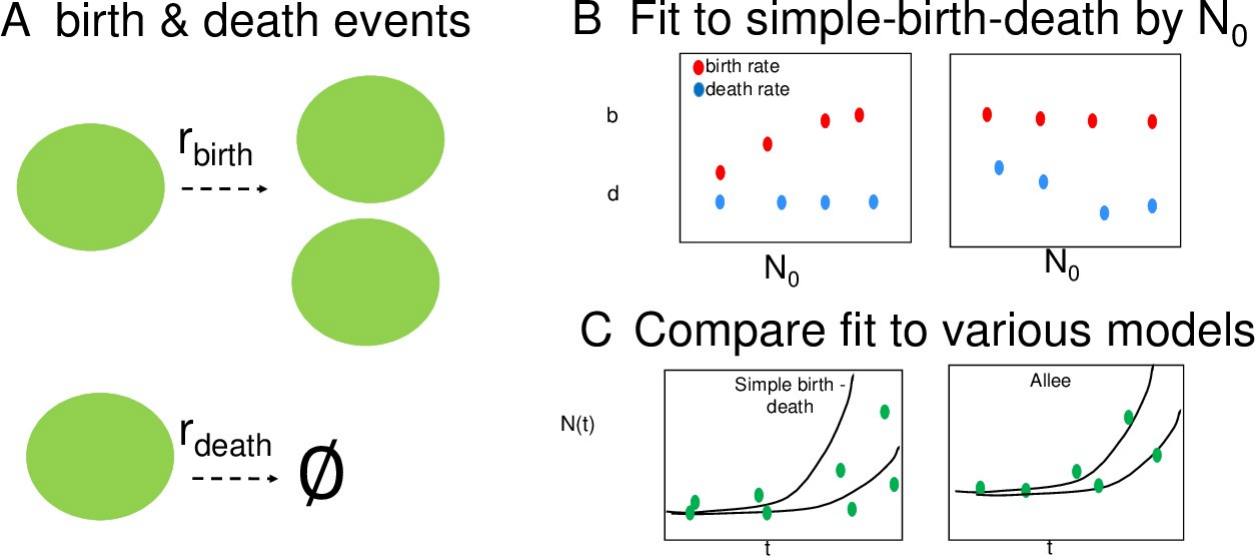
A stochastic model of tumor growth and expected outputs if Allee effect is present. (A) Schematic illustration of generalized stochastic framework in which a cell can either give birth or die at a rate given by *r*_*birth*_ or *r*_*death*_ respectively. (B) Schematic of the expected results from fitting of the simplest birth–death model with each data set grouped by initial cell number (N_0_), where, if an Allee effect is present, we expect to observe that either the birth rate constant (*b*) (red) or death rate constant (*d*) (blue) change with the initial condition. (C) Schematic of the expected outcomes of fitting full data set to the simple birth–death model (left) and a model incorporating an Allee effect (right).

We developed 7 stochastic models whose expected mean cell number in time ⟨*n*(*t*)⟩ are equivalent to that predicted by the deterministic models described above (Eqs 1, 4 and 5). For each deterministic model structure, the total number of cells *n*(*t*) is modeled by the ODEs in Eqs 1, 4 and 5 above. The time evolution of *n*(*t*) for the stochastic models are defined by the following birth and death events (Fig 2A):

$$\mathrm{Event}\;1:\;\mathrm{birth}\;C\to 2C(\mathrm{reaction}\;\mathrm{rate}:r_{\mathrm{birth}}(n))$$

$$\mathrm{Event}\;2:\;\mathrm{death}\;C\to \emptyset (\mathrm{reaction}\;\mathrm{rate}{:r}_{\mathrm{death}}(n))$$

Where *r*_birth_(*n*) and *r*_death_(*n*) describe the rate at which the events occur, which may depend on the number of cells, *n*, present or be constant. The probability of an event *i* happening in an infinitesimal time step *Δt* is given generally by the product of the rate of the event, the state of the population, and the time step:

$$P_{event}=r_{event}(n)n\Delta t$$ (6)

For our purposes in modeling tumor growth, we limit the possible events to birth or death events, and in all cases, the probability of an event occurring is a function of *n*, because it is a first-order reaction described by the schematic in (Fig 2A).

For each model presented, the generalized framework described above holds and only the birth and death rates (r_birth_ and r_death_) differ for each model based on the hypothesis about the dependency of the birth or death rate on cell number. To give an illustrative example of the components of the stochastic model, we explicitly state the reaction rates and the resulting birth and death probabilities for the simple birth–death model. To remain concise, for the remaining 6 Allee models described, we just present the birth and death probabilities for each model.

In the simple birth–death model, the birth rate and death rates are independent of cell number, *n*. They are described by rate constants, denoted *b* and *d*:

$$r_{birth}=b$$ (7)

$$r_{death}=d$$ (8)

And birth and death probabilities in time step *Δt* of

$$P_{birth}=bn\Delta t$$ (9)

$$P_{death}=dn\Delta t$$ (10)

The average behavior in this model corresponds to the exponential growth model (Eqs 1 and 2), where the growth rate constant *g* is equivalent to the birth rate constant (*b*) minus the death rate constant (*d*) (*g* = *b* − *d*). For the remaining stochastic models we introduce birth and/or death rates that are functions of *n*, corresponding to the hypotheses that the birth and/or death rates are not constant and instead depend on the population size, *n*. Similar to the way that stochastic growth models have modified the growth rate with a term that decreases growth rate in proportion to increasing *n* (i.e., in the work by Sun and colleagues [43], where the division rate *k* is defined as *k* = *k*_0_−*γn*), we prescribe modifications to birth and death rates that decrease birth rates proportional to the reciprocal of *n*. We note that this nonlinear dependency is prescribed as such in order to achieve the desired slowing of growth rates at low *n* that the Allee effect models produce (Eqs 4 and 5). The first stochastic Allee model is the strong Allee on birth model.

$$P_{birth}=(b-\frac{A}{n}(b-d))n\Delta t;$$ (11)

$$P_{death}=dn\Delta t.$$ (12)

This model hypothesizes that the birth probability is lowered by a factor proportional to the growth rate (*b* − *d*) and the reciprocal of *n*, and thus for large *n*, the $\frac{A}{n}$ term in Eq 12 is negligible, but at small *n* the birth probability is significantly decreased by the Allee term, resulting in a lower birth probability and observed slower net growth.

Alternatively, we can hypothesize that the Allee effect acts to increases the death probability at *n* near *A*, resulting in the strong Allee on death model probabilities of

$$P_{birth}=bn\Delta t;$$ (13)

$$P_{death}=(d+\frac{A}{n}(b-d))n\Delta t.$$ (14)

And lastly, we present a model that assumes that the Allee effect term acts equally on both decreasing the birth probability and increasing the death probability for *n* near *A*, resulting in

$$P_{birth}=(b-\frac{A}{n}\frac{\left(b-d\right)}{2})n\Delta t;$$ (15)

$$P_{death}=(d+\frac{A}{n}\frac{\left(b-d\right)}{2})n\Delta t.$$ (16)

For simplicity, this model assumes that the Allee term acts equally, with half of its effect decreasing the birth rate and half increasing the death rate at *n* near *A*. Of course, there could be an infinite number of ways of distributing the Allee threshold onto the birth and death probabilities, and this could have been introduced with an additional fractional parameter. However, for simplicity, we only consider equal partitioning of the Allee effect on both birth and death rates.

The last family of stochastic models corresponds to the extended Allee model (Eq 5). Again, this model introduces birth and death rate dependencies on *n*. By the same arguments described for the strong Allee effect model, the extended Allee effect model can manifest itself either on the birth probability only, the death probability only, or the birth and death probabilities equally, leading to the following birth and death probabilities.

If the Allee effect acts on birth only:

$$P_{birth}=(b-(b-d)\frac{A+\tau }{n+\tau })n\Delta t;$$ (17)

$$P_{death}=dn\Delta t.$$ (18)

If the Allee effect acts on death only:

$$P_{birth}=bn\Delta t;$$ (19)

$$P_{death}=(d+(b-d)\frac{A+\tau }{n+\tau })n\Delta t.$$ (20)

If the Allee effect term acts on birth and death equally:

$$P_{birth}=(b-\frac{\left(b-d\right)}{2}\frac{A+\tau }{n+\tau })n\Delta t;$$ (21)

$$P_{death}=(d+\frac{\left(b-d\right)}{2}\frac{A+\tau }{n+\tau })n\Delta t.$$ (22)

A complete description of each of the above 7 stochastic models grouped by the corresponding deterministic model and their assumptions of birth or death mechanism is displayed in Table 1.

**Table 1:** Stochastic growth model families whose average behavior correspond to one of the deterministic growth models. For the Allee model families, within each family, the Allee effect can alter birth, death, or both probabilities, representing distinct mechanistic hypotheses.

Exponential model family	Strong Allee model family	Extended Allee model family
**Mean cell-number change expressed as a deterministic ODE of each family of stochastic models**		
$\frac{\partial N(t)}{\partial t}=gN(t)$	$\frac{\partial N(t)}{\partial t}=g(1-\frac{A}{N(t)})N(t)$	$\frac{\partial N(t)}{\partial t}=g(1-\frac{A+\tau }{N(t)+\tau })N(t)$
**Probabilities of birth and death events to describe stochastic models within each family**		
*P*_*birth*_ = *Δt*(*bN*) *P*_*death*_ = *Δt*(*dN*)	**Allee effect on birth rate**	**Allee effect on birth rate**
	*P*_*birth*_ = *Δt*(*bN*−(*b*−*d*)*A*) *P*_*death*_ = *Δt*(*dN*)	$P_{birth}=\Delta t(bN-(b-d)N(\frac{A+\tau }{N+\tau }))$ *P*_*death*_ = *Δt*(*dN*)
	**Allee effect on death**	**Allee effect on death**
	*P*_*birth*_ = *Δt*(*bN*) *P*_*death*_ = *Δt*(*dN*+(*b*−*d*)*A*)	*P*_*birth*_ = *Δt*(*bN*) $P_{death}=\Delta t(dN+(b-d)N(\frac{A+\tau }{N+\tau }))$
	**Allee effect on birth and death**	**Allee effect on birth and death**
	$P_{birth}=\Delta t(bN-(\frac{\left(b-d\right)}{2})A)$ $P_{death}=\Delta t(dN+(\frac{\left(b-d\right)}{2})A)$	$P_{birth}=\Delta t(bN-N(\frac{b-d}{2})(\frac{A+\tau }{N+\tau }))$ $P_{death}=\Delta t(dN+N(\frac{b-d}{2})(\frac{A+\tau }{N+\tau }))$

**Abbreviation:** ODE, ordinary differential equation

To simulate growth trajectories of the stochastic models, we use the Gillespie algorithm (S1 Text) [44, 45]. The above models are used to test the relevance of the Allee effect in cancer cell population growth. The conventional exponential growth model (Eqs 1 and 2) assumes that growth rate (birth rate minus death rate) is constant and independent of initial condition. To test the validity of this assumption in an exploratory analysis, we first fit each group of trajectories individually for each initial cell number, N_0_. If an Allee effect is present in the data, a systematic increase in the fitted birth rate constant, *b*, or decrease in death rate constant, *d*, with increasing initial cell number would be expected (shown schematically in Fig 2B). We next investigated the relevance of the 7 stochastic models by fitting the simulated cell-number trajectories from all initial conditions to each stochastic model described above (Eqs 9–22) to determine which model structure best describes the observed growth dynamics (shown schematically in Fig 2C).

### Parameter estimation and model selection framework

**Fig 3:**
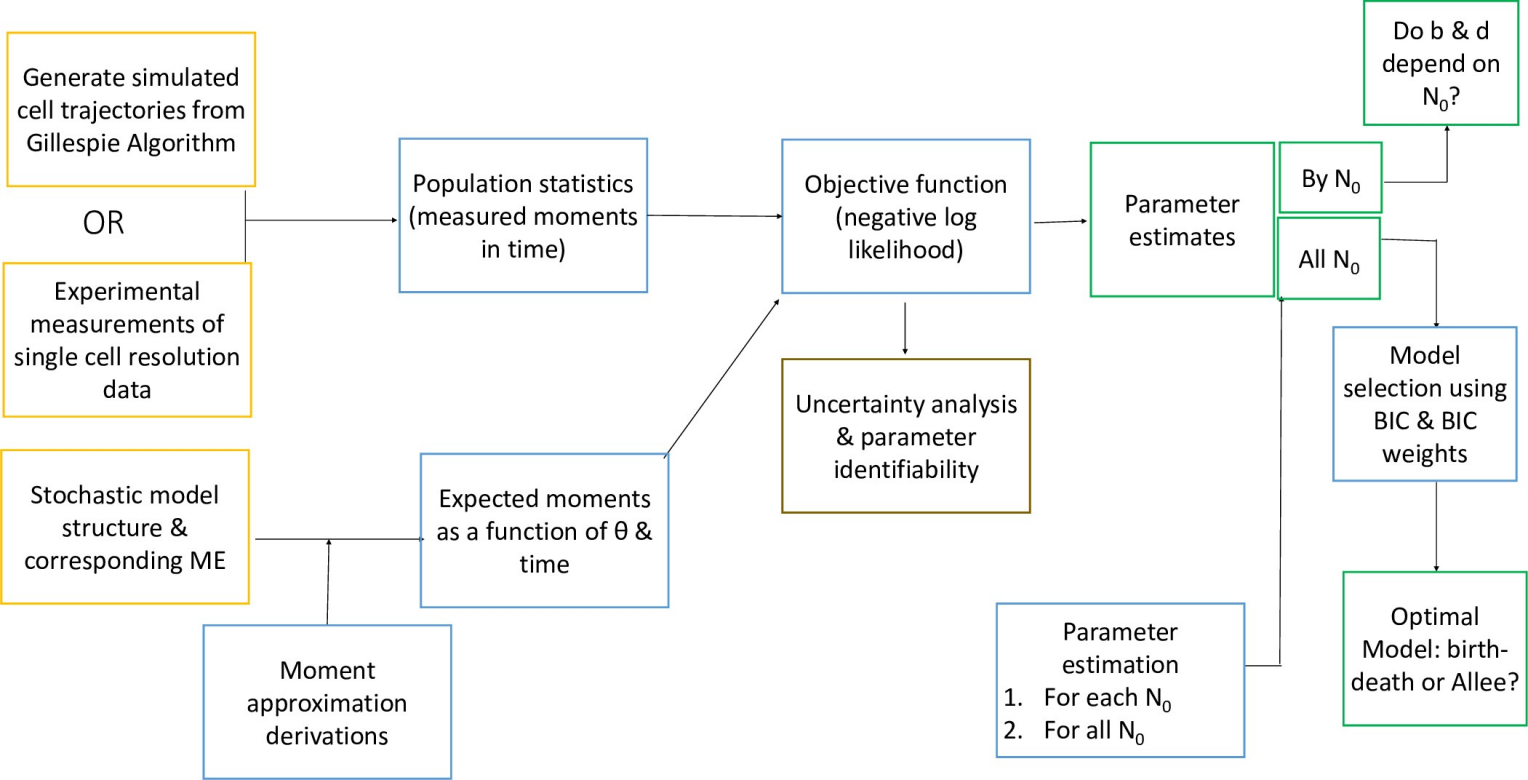
Moment-closure approximation approach for stochastic parameter estimation. Framework for moment-closure approximation approach to derive moments from the ME of a stochastic process and how model expected moments are fit to stochastic data. BIC, Bayesian Information Criterion, ME, master equation.

The parameters of stochastic processes are often inferred using approximate Bayesian computation [46], which require exhaustive stochastic simulations in order to minimize the differences between simulation and experimental data for each parameter set searched. These algorithms require a high number of simulating runs, making them computationally expensive and instantiating issues of nonconvergence and model selection [47]. To render inference on the stochastic process feasible, we apply the moment-closure approximation method described in Frohlich and colleagues [42] to fit the 7 proposed stochastic growth models to experimentally measured growth curves (Fig 3).

#### The master equation of a stochastic process

The master equation (ME) describes the change in the probability distribution that the system has (in this case number of cells, *n*) as a function of time. From the ME, the time derivative of the moments, or expected values of *n*, *n*_2_,…*n*_*m*_ can be derived. In this framework, we developed stochastic models so that the derivative of the first-order moment corresponds to one of the deterministic models presented (Eqs 2, 4 and 5). The ME describes the probability of their being *n* cells at time *t* as a sum of probabilities of a birth, death, or neither event occurring given there are *n* − 1, *n* + 1, and *n* cells at time *t*, respectively:

$$\frac{\partial p_{n}(t)}{\partial t}=r_{birth}(n-1)p_{n-1}(t)-[r_{death}(n)+r_{birth}(n)]p_{n}(t)+r_{death}(n+1)p_{n+1}(t),$$ (23)

where r_birth_ and r_death_ are functions of the parameters *b*, *d*, *A* and/or τ for each of the 7 stochastic structural models (S2 Table).

#### Derivations of moment-closure approximations from the ME

From longitudinal data of cell number over time (*N*(*t*)), with sufficient replicates, we expect to be able to measure the mean and variance in cell number over time. We want to be able to directly compare the mean and variance in the experimental longitudinal data to the model expected mean and variance in time as a function of the model parameters. We therefore want to derive the first and second moments from the ME. From the ME of each stochastic model (S2 Table), the time derivative of the first and second moments were derived according to the procedure outlined in [48](S2 Text). Using the definition of variance, the ODEs of the mean and variance for each model can be written in terms of the lower order moments (⟨***n***⟩ and ⟨***n***^2^⟩), where ⟨…⟩ denotes the expectation value of the moment; Table 1). Within each family of models (exponential, strong Allee, and extended Allee; Eqs 2, 4 and 5) the stochastic forms (Eq 9–22) share the same mean ODE corresponding to their deterministic model family but differ in their variance based on whether the Allee effect alters the birth, death, or both event terms. The time evolution of the variance can be used to properly identify individual rate parameters such as the birth and death rates because the variance in time is proportional not just to the net growth (birth minus death rates) but to the sum of the birth and death rates, as shown in S1 Fig.

For each stochastic model, we confirmed that the mean and variance of a simulated data set of 5,000 trajectories with known parameters matched the derived model mean and variance described in Table 2. (See S3 Text, S2–S8 Figs)

**Table 2:** Differential equations of the moment-closure approximations of the mean and variance for each stochastic model obtained from the ME using the moment approach.

Model	Mean ⟨*n*(*t*)⟩	Variance ⟨*Σ*(*t*)⟩
**Simple birth-death**	$\frac{\partial \langle n\rangle }{\partial t}=\langle n\rangle (b-d)$	$\frac{\partial \langle \Sigma_{ii}\rangle }{\partial t}=2(b-d)\langle \Sigma_{ii}\rangle +(b+d)\langle n\rangle$
**Strong Allee on birth**	$\frac{\partial \langle n\rangle }{\partial t}=\langle n\rangle (b-d)(1-\frac{A}{\left\langle n\right\rangle })$	$\frac{\partial \langle \Sigma_{ii}\rangle }{\partial t}=2\langle n^{2}\rangle (b-d)-2\langle n\rangle (b-d)A+(b+d)\langle n\rangle -(b-d)A-2\langle n\rangle (b-d)(\langle n\rangle -A)$
**Strong Allee on death**	$\frac{\partial \langle n\rangle }{\partial t}=\langle n\rangle (b-d)(1-\frac{A}{\left\langle n\right\rangle })$	$\frac{\partial \langle \Sigma_{ii}\rangle }{\partial t}=2\langle n^{2}\rangle (b-d)-2\langle n\rangle (b-d)A+(b+d)\langle n\rangle +(b-d)A-2\langle n\rangle (b-d)(\langle n\rangle -A)$
**Strong Allee on both**	$\frac{\partial \langle n\rangle }{\partial t}=\langle n\rangle (b-d)(1-\frac{A}{\left\langle n\right\rangle })$	$\frac{\partial \langle \Sigma_{ii}\rangle }{\partial t}=2\langle n^{2}\rangle (b-d)-2\langle n\rangle (b-d)A+(b+d)\langle n\rangle -2\langle n\rangle (b-d)(\langle n\rangle -A)$
**Extended Allee on birth**	$\frac{\partial \langle n\rangle }{\partial t}=\langle n\rangle (b-d)(1-\frac{A+\tau }{\langle n\rangle +\tau })$	$\frac{\partial \langle \Sigma_{ii}\rangle }{\partial t}=2\langle n^{2}\rangle (b-d)+\langle n\rangle (b+d)-2\langle n^{2}\rangle (b-d)(\frac{A+\tau }{\langle n\rangle +\tau })-\langle n\rangle (b-d)(\frac{A+\tau }{\langle n\rangle +\tau })-2\langle n^{2}\rangle (b-d)(1-\frac{A+\tau }{\langle n\rangle +\tau })$
**Extended Allee on death**	$\frac{\partial \langle n\rangle }{\partial t}=\langle n\rangle (b-d)(1-\frac{A+\tau }{\langle n\rangle +\tau })$	$\frac{\partial \langle \Sigma_{ii}\rangle }{\partial t}=2\langle n^{2}\rangle (b-d)+\langle n\rangle (b+d)-2\langle n^{2}\rangle (b-d)(\frac{A+\tau }{\langle n\rangle +\tau })+\langle n\rangle (b-d)(\frac{A+\tau }{\langle n\rangle +\tau })-2\langle n^{2}\rangle (b-d)(1-\frac{A+\tau }{\langle n\rangle +\tau })$
**Extended Allee on both**	$\frac{\partial \langle n\rangle }{\partial t}=\langle n\rangle (b-d)(1-\frac{A+\tau }{\langle n\rangle +\tau })$	$\frac{\partial \langle \Sigma_{ii}\rangle }{\partial t}=2\langle n^{2}\rangle (b-d)+\langle n\rangle (b+d)-2\langle n^{2}\rangle (b-d)(\frac{A+\tau }{\langle n\rangle +\tau })-2\langle n^{2}\rangle (b-d)(1-\frac{A+\tau }{\langle n\rangle +\tau })$

**Abbreviation:** ME, master equation

#### Maximum likelihood and Bayesian parameter estimation

To infer the parameters of the stochastic models, a maximum likelihood parameter estimation approach was employed using derivations from Frohlich and colleagues [42]. The likelihood function assumes that the measured mean and variance of the data at each time point *t*_*k*_ is normally distributed around the model predicted first moment, mean cell number (*μ*_*i*_(*t*_*k*_,*θ*)) and mean variance in cell number (Σ_*ii*_(*t*_*k*_,*θ*)) with standard deviations for each distribution of mean cell number and variance in cell number given by $\sigma_{\mu_{i,k}}(\theta )$ and $\sigma_{\Sigma_{ii,k}}(\theta )$ respectively. These standard deviations in the first moment and variance, *σ*_*μ*_(*θ*) and *σ*_*Σ*_(*θ*), are functions of the parameters *θ* and were derived by Frohlich and colleagues [42]. The likelihood function (Eq 24) and its corresponding negative log likelihood (Eq 25) are the following:

$$L(\theta )=\prod_{i,k}\frac{1}{\sqrt{2\pi \sigma_{\mu_{i,k}}^{2}(\theta )}}\exp(-\frac{1}{2}(\frac{\mu_{i}(t_{k},\theta )-{\hat{u}}_{i,k}}{\sigma_{\mu_{i,k}}(\theta )}{)}^{2})\times \prod_{i,k}\frac{1}{\sqrt{2\pi \sigma_{\Sigma_{ii,k}}^{2}(\theta )}}\exp(-\frac{1}{2}(\frac{\Sigma_{ii,k}(t,\theta )-{\hat{\Sigma }}_{ii,k}}{\sigma_{\Sigma_{ii,k}}(\theta )}{)}^{2});$$ (24)

$$NLL(\theta )=\frac{1}{2}\sum_{k,i}\left(\log\;2\pi \sigma_{\mu_{i,k}}^{2}(\theta )+(\frac{\mu_{i}(t_{k},\theta )-{\hat{u}}_{i,k}}{\sigma_{\mu_{i,k}}(\theta )}{)}^{2}\right)+\frac{1}{2}\sum_{k,i}\left(\log\;2\pi \sigma_{\Sigma_{ii,k}}^{2}(\theta )+(\frac{\Sigma_{ii,k}(t,\theta )-{\hat{\Sigma }}_{ii,k}}{\sigma_{\Sigma_{ii,k}}(\theta )}{)}^{2}\right).$$ (25)

These weigh equally the likelihood of the measured mean and variance of the data from each trajectory over all time points measured. To perform maximum likelihood parameter estimation, we used the fminsearch function in MATLAB to minimize the NLL(*θ*) (Eq 25). For this optimization, non-negative parameters (rate constants *b* and *d*) were log-transformed while parameters allowed to be negative (extended Allee model Eqs 17–22 parameters *A* and *τ*) were normalized between 0 and 1 over a domain of reasonable values of *A* and *τ*. We used the log of the slope of the mean cell number in time as an initial guess for the growth rate (*b* − *d*), a death rate of *d* = 0.0005 cells/hour, *A* = 1 or −1 and *τ* = 2 were used in order to make a conservative initial guess.

#### Uncertainty analysis and parameter identifiability

A key benefit of the moment approach for stochastic parameter estimation is that deriving a system of coupled differential equations enables the use of already established methods for parameter identifiability and uncertainty analysis. To evaluate structural identifiability from each model, a differential algebra approach [49, 50] was used to reveal identifiable combinations of parameters in terms of the output we were able to measure in time—in this case, both the mean and the variance of the cell-number trajectories in time. (See S4 Text for an example of this approach applied to the birth–death model). This analysis revealed that the parameters in all 7 models are uniquely identifiable.

To ensure that the predicted mean and variance of the models exhibited distinguishable differences from each other qualitatively, we investigated some illustrative cases of the expected mean and variance for a simple birth–death model, a strong Allee model, and a weak case of the extended Allee model (S9A and S9B Fig). Likewise, to ensure the different forms of the stochastic models within each broader class of deterministic models were distinguishable by the expected differences in their variance (Table 1), we display the solutions of the expected mean and variance for strong and weak Allee effects on both birth, death, and equally on both (S10A–S10D Fig). This gave us confidence that the candidate models were theoretically distinguishable using the mean and variance of the data collected. To evaluate whether these model parameters were practically identifiable and quantify the corresponding uncertainty on these model parameters, the profile likelihood method was used as described in [51]. The profile likelihood method evaluates the ability to uniquely identify each parameter individually by profiling one parameter at a time, fixing it to a range of values, and fitting for the rest of the parameters at each fixed value. The resulting curvature of likelihood is used to evaluate the uncertainty on the parameter and determine confidence intervals.

### Modeling framework is able to distinguish between different growth models from simulated stochastic trajectories

**Fig 4:**
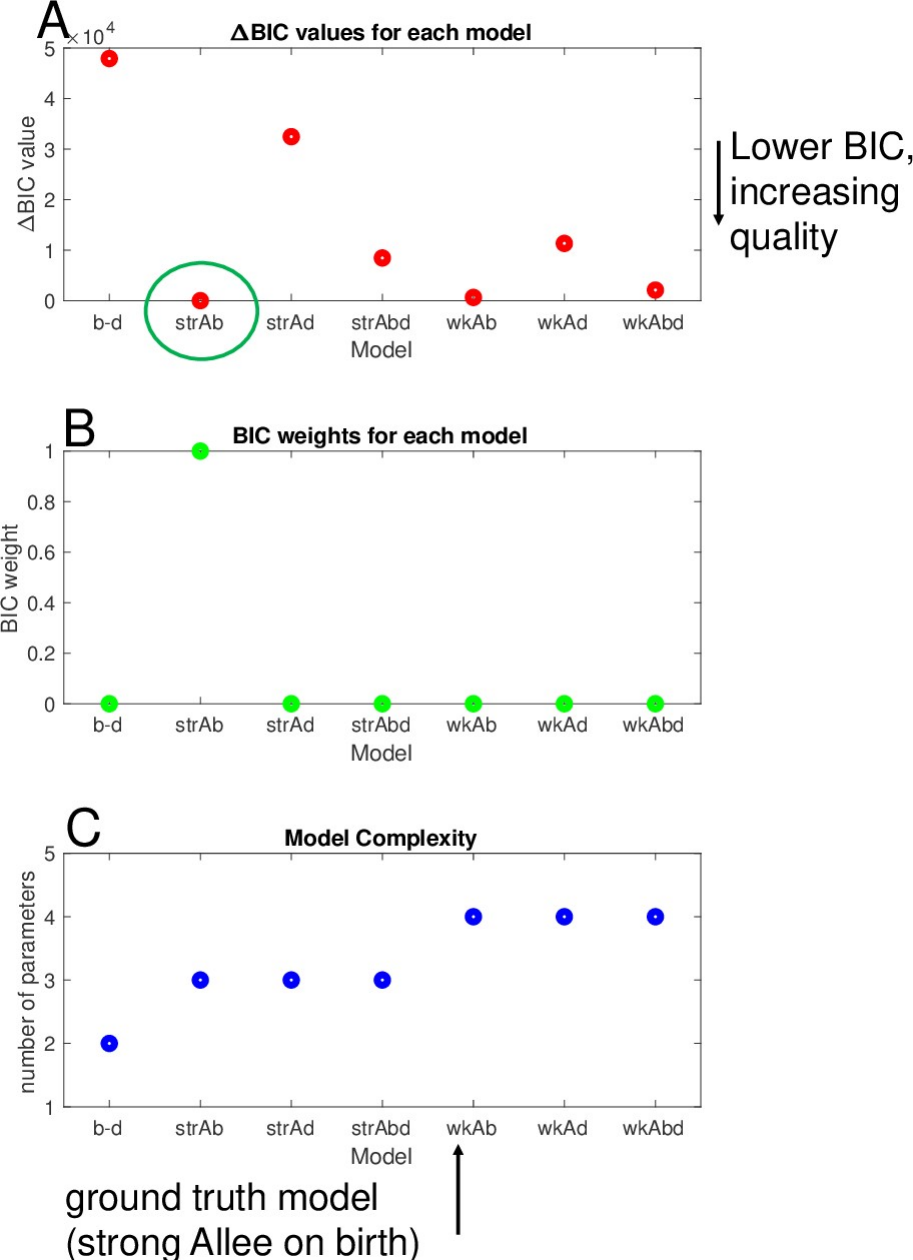
Model selection based on the BIC identifies the ground truth model in simulated data. (A) ΔBIC values (*BIC*_*i*_−*BIC*_*min*_) are plotted for the fit of the simulated data set to each of the 7 models from left-to-right: simple birth–death model (*b* − *d*), strong Allee model on birth (str*Ab*), strong Allee model on death (str*Ad*), strong Allee model on birth and death (str*Abd*), weak extended Allee model on birth (wk*Ab*), weak extended Allee model on death (wk*Ad*), and weak extended Alee model on birth and death (wk*Abd*) compared with the highest quality, minimum BIC value model: the strong Allee model on birth. (B) BIC weighting reveals strong evidence to choose the strong Allee model over the other candidate models. (C) Number of parameters of each model as a measure of relative complexity of the model. The data and code used to generate this figure can be found at https://github.com/brocklab/Johnson-AlleeGrowthModel.git. BIC, Bayesian Information Criterion.

**Fig 5:**
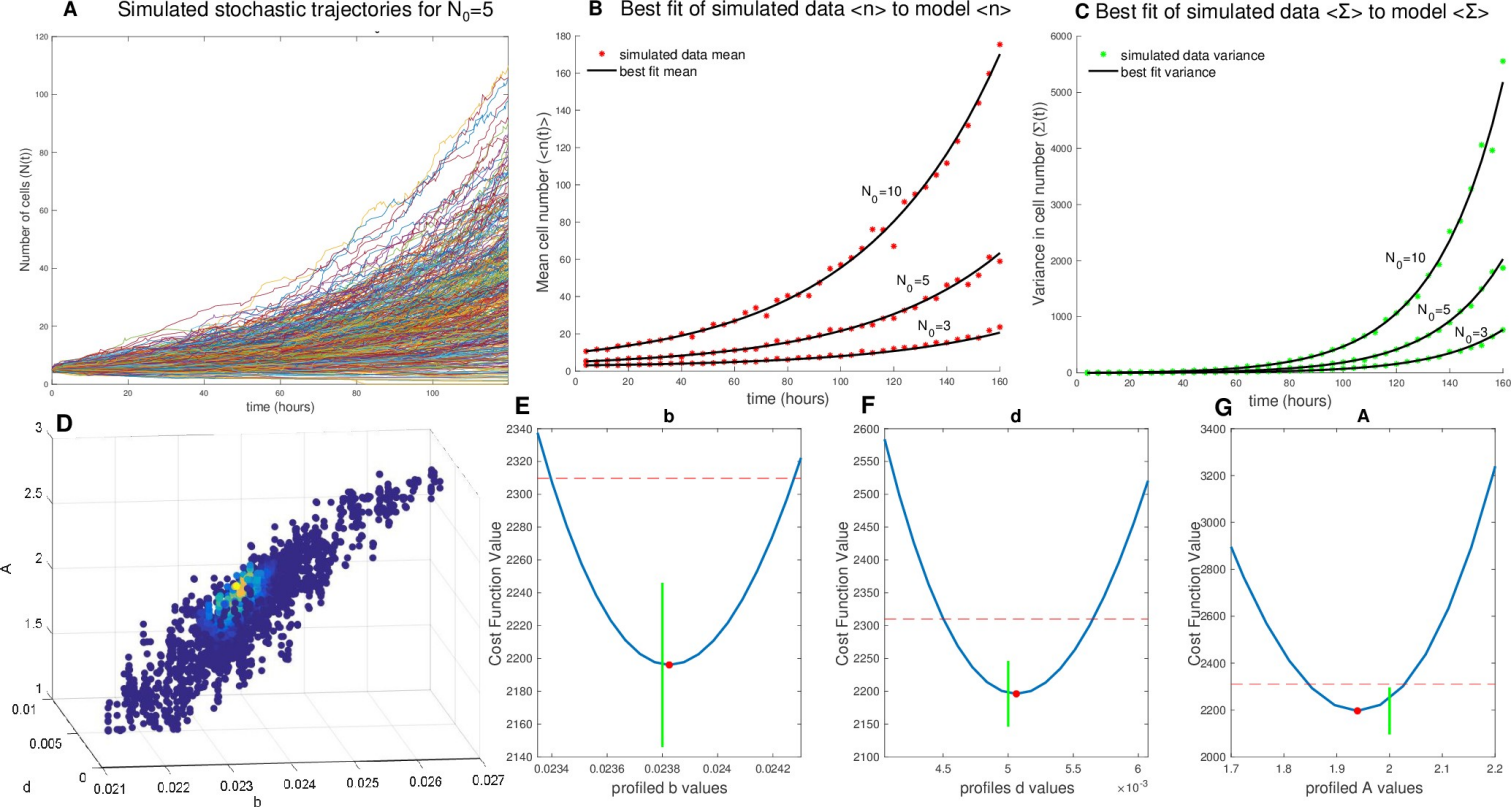
Fit to mean and variance from simulated stochastic data set. (A) Example of stochastic growth model output from 5,000 simulated cell-number trajectories with initial condition of N_0_ = 5 and a birth rate of *b* = 0.0238, death rate of *d* = 0.005, and an Allee threshold *A* = 2, revealing the expected variability in growth dynamics apparent at low initial numbers. (B) From the simulated stochastic trajectories, we sample time uniformly and measure the mean cell number at each time point for N_0_ = 3, 5, and 10. (C) Again from the simulated stochastic trajectories, we sample time uniformly and measure the variance in cell number at each time point for N_0_ = 3, 5, and 10 (D) Display of parameter space searched, with parameter sets of *b*, *d*, and *A* colored by likelihood, indicating the framework converges on the true parameters. (E) Profile likelihood analysis of birth rate parameter estimate (red dot) of *b* = 0.0238 [0.02340–0.02425] with true *b* = 0.0238 (green line). (F) Profile likelihood analysis of death rate parameter estimate (red dot) *d* = 0.0051 [0.00461–0.00563] with true *d* = 0.005 (green line). (G) Profile likelihood analysis of Allee threshold parameter estimate (red dot) of *A* = 1.9393 [1.853–2.026] with true *A* = 2 (green line). The data and code used to generate this figure can be found at https://github.com/brocklab/Johnson-AlleeGrowthModel.git.

The parameter estimation and model selection framework were verified by applying the calibration scheme to simulated data from a model of intermediate complexity—the strong Allee effect on birth (Eqs 11 and 12). Using the Gillespie algorithm [44, 45], we generated 5,000 simulated trajectories from initial conditions of N_0_ = 3, 5, and 10 from the strong Allee effect on birth model. In order to most closely simulate the expected experimental data, the stochastic trajectories were sampled every 4 hours corresponding to the time intervals used in the experimental measurements of cell growth, and the mean and variance were calculated at each time point. A constant random noise term was added to the measurements of mean and variance in time in order to generate trajectories that resembled experimental measurements of mean and variance and to simulate the additive noise of the experimental system (Fig 4A and 4B). The simulated data were fit to the 7 candidate models (Eqs 9–22, Table 2) representing the range of biological hypotheses, with model complexities ranging from 2 to 4 parameters. To identify the most likely underlying model structure from each of the candidate stochastic models, the Bayesian Information Criterion (BIC) was used for model selection [52–54] (See S5 Text). As expected, the strong Allee effect on birth had the lowest BIC value (Fig 4A), revealing that the underlying model structure was correctly identified. The BIC weighting analysis [54] (S5 Text) revealed strong evidence in favor of the strong Allee effect on birth (Fig 4B), indicating the ability of the BIC value to distinguish between overly simple models with 2 parameters and overly complex models with 4 parameters (Fig 4C). In order to ensure the method was not overweighing goodness of fit, the data were down-sampled from the true data collection interval of every 4 hours to every 36 hours to demonstrate that down-sampling changed the magnitudes of the BIC values but did not affect the order of the BIC values of each model relative to one another (S11 Fig). The chosen model provided a good fit to the mean and variance in the data (Fig 5B and 5C), and the parameter search displayed the expected convergence of accepted parameter values (Fig 5D). Profile likelihoods on parameter distributions demonstrated that each of the parameters were practically identifiable and parameter estimates fell close to the true parameters (Fig 5E, 5F and 5G). The true parameter values of *b* = 0.00238, *d* = 0.0005, and *A* = 2 fell within the confidence intervals of the profile likelihood analysis of the fitted parameters of [0.02340–0.02425] for *b*, [0.00461–0.00563] for *d*, and [1.853–2.026] for *A*. This confirms that the calibration approach selects the appropriate underlying model structure from a set of hypotheses and properly identifies the parameters.

### Experimental measurement reveals scaling of growth rate with initial cell number

**Fig 6:**
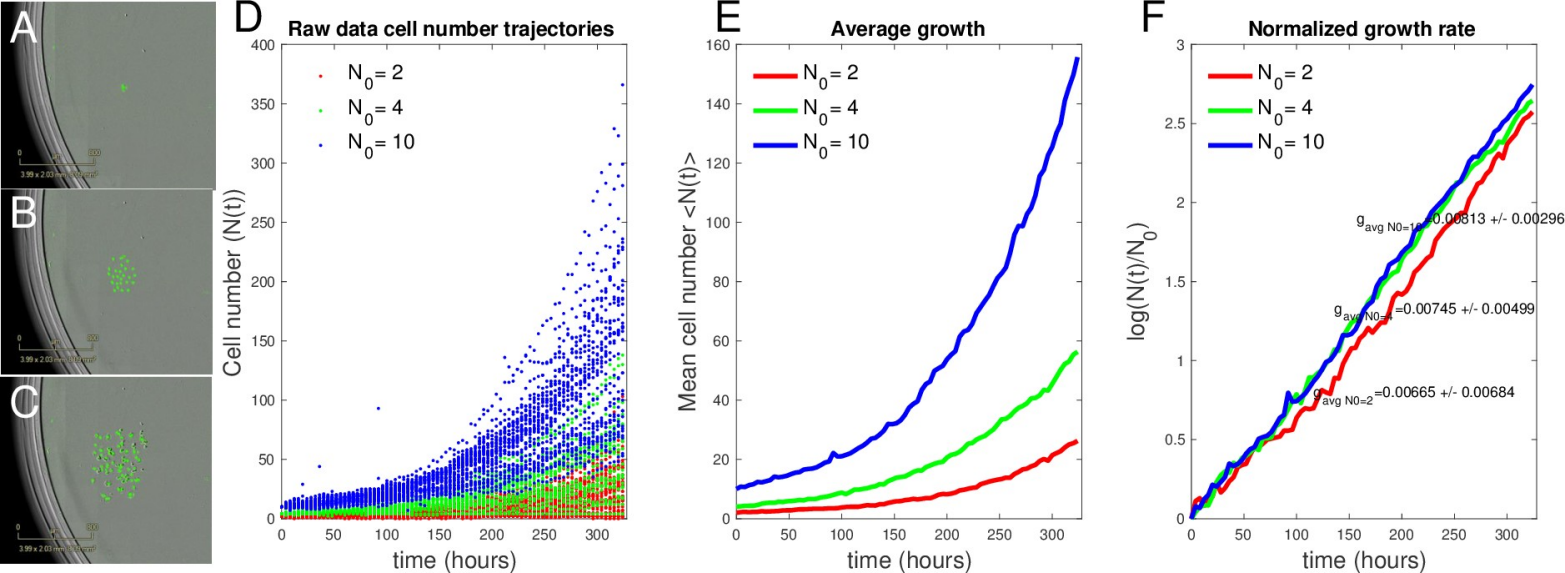
BT-474 cancer cells in culture exhibit growth rate scaling with initial cell density. (A, B, and C) Representative images from day 1 (A), day 6 (B), and day 14 (C) of BT-474 GFP labeled cells proliferating in vitro. (D) Individual cell-number trajectories for different N_0_ = 2, 4, and 10. (E) Average cell number every 4 hours from each trajectory of N_0_ = 2, 4, and 10. (F) Cell number in time normalized by initial cell number in log scale reveals scaling of growth rate by initial cell number, with *g* = 0.00665 ± 0.00684, 0.00745 ± 0.00499, and 0.00813 ± 0.00296 for N_0_ = 2, 4, and 10, respectively.The data and code used to generate this figure can be found at https://github.com/brocklab/Johnson-AlleeGrowthModel.git.

Next, we investigated whether the growth of cancer cells in vitro is governed by alternative growth models other than the exponential growth model commonly used to describe tumor cell growth well below carrying capacity. BT-474 breast cancer cells were seeded at a precise initial cell number ranging from 1 to 20 cells per well of a 96-well plate, and time-lapse microscopy images were collected every 4 hours for replicate wells at each initial condition (20–50 wells per condition; see Cell culture and low cell density seeding). Example images are shown in Fig 6A, 6B and 6C. Cell number as function of time was measured for a total of 328 hours (just under 2 weeks) and cell-number counts in time were determined using digital image processing for each individual well imaged (see Time-lapse imaging and image analysis).

The true initial cell number (N_0_) sorted into each well was confirmed by eye from the initial image, and wells were binned according to the observed initial cell number. Cell-number trajectories of wells with initial cell numbers of 2, 4, and 10 cells are shown in Fig 6D in red, green, and blue, respectively. As a preliminary analysis of this data, we fitted each well individually to the exponential growth model (Eqs 1 and 2) to obtain a distribution of growth-rate constants at each initial condition. The mean growth rates for *N*_*0*_ = 2, 4, and 10, respectively, were *g* = 0.00665 ± 0.00684, 0.00745 ± 0.00499, and 0.00813 ± 0.00296. Fig 6 displays the average cell-number trajectory (Fig 6E) and the normalized growth rate ($log(\frac{N(t)}{N_{0}})$); Fig 6F) for the measured data at each time point. These results indicate clear deviations from the simple exponential growth model in which the normalized growth rate ($log(\frac{N(t)}{N_{0}})$) is expected to be identical for all initial conditions (see Fig 1C). Instead, growth behavior resembled the characteristic scaling of normalized cell numbers by initial cell number that is observed for both Allee effect models (Fig 1F and 1I). The scaling of average growth rate with initial cell number had been observed by Neufeld and colleagues [3] in their in vitro studies of cell culture, providing us with the motivation to further investigate whether an Allee model better describes BT-474 breast cancer cell growth.

To ensure that the observed differences in growth rate at low cell densities were significantly different from what is observed at normal cell culture seeding densities, we sorted N_0_ = 512 and N_0_ = 1,024 cells and captured 30 growth trajectories from each initial condition. The mean and standard deviation of the growth rates were not significantly different from one another and also significantly higher than the observed low cell density growth rates, with *g* = 0.0112 ± 0.00062 and *g* = 0.0115 ± 0.00074 for N_0_ = 512 and N_0_ = 1,024, respectively (S12 Fig). The absence of density-dependent growth rates at these higher initial cell numbers may explain why the Allee effect hasn’t been described using standard cell culture seeding densities.

### Fit of experimental data to stochastic growth models reveals Allee effect

The variability in the observed cell-number trajectories for a single initial condition is reflected in the experimental measurements of BT-474 cells growing at low initial cell densities (Fig 6D). This variability in cell growth dynamics is expected due to the inherent stochasticity of the birth and death processes, which is apparent at the small population sizes measured in this study (Fig 5A). Because stochasticity is more apparent and can be observed experimentally at the low cell numbers (Fig 6D), such dynamics are appropriately modeled by a stochastic rather than a deterministic process. In order to determine whether the preliminary observations of growth-rate scaling with the initial cell number could be described by alternative models of cell population growth that consider the Allee effect, the experimental data of BT-474 growth trajectories shown in Fig 6D for initial cell numbers of 2, 4, and 10 were calibrated to the 7 stochastic models using the stochastic modeling framework presented above (Fig 3).

### Fitting each initial condition separately to the simple birth–death model reveals net growth rate increases with initial cell number

**Fig 7:**
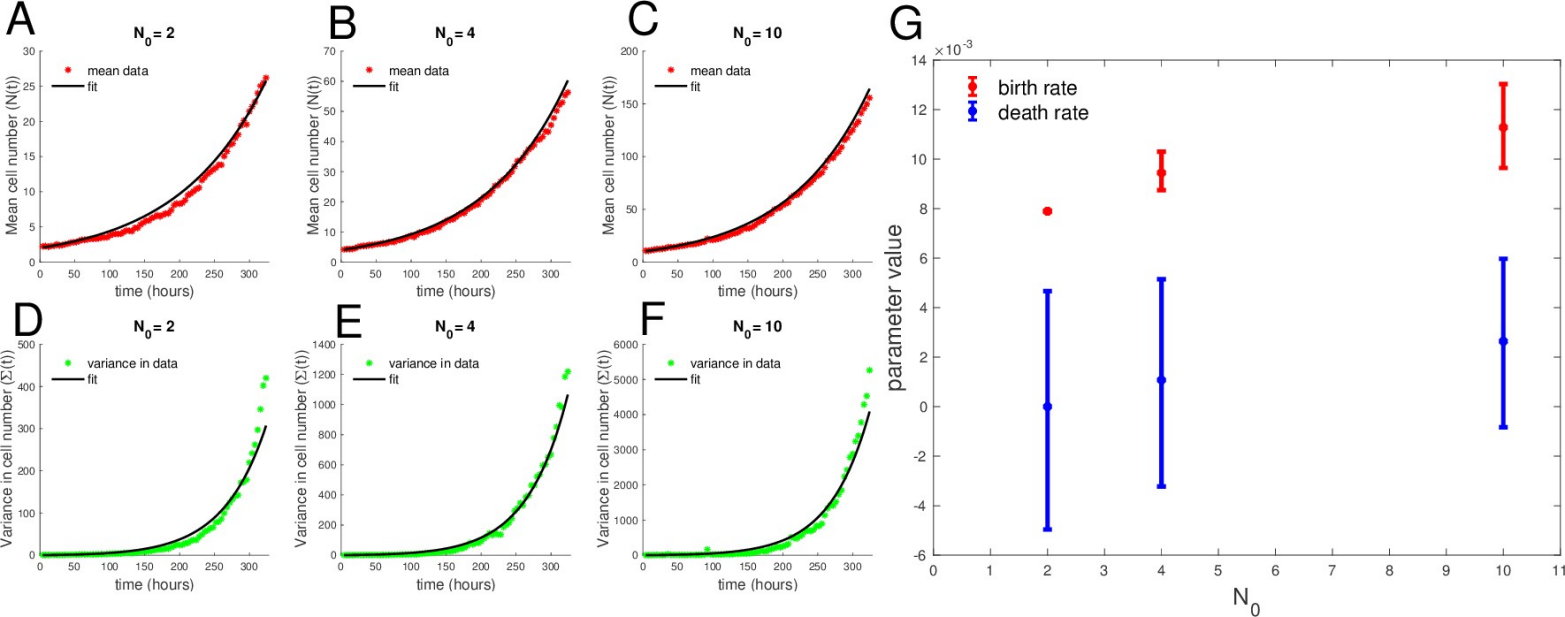
Best fit of each initial cell-number means and variances in time to stochastic birth–death model reveals net growth rate increases with initial cell number. (A, B, and C) Data mean over time compared to best fit to model mean. (D, E, and F) Data variance in time compared to best fit to model variance. (G) Best fit birth and death rate parameters for the stochastic birth–death model fit to each initial condition, with confidence intervals determined from profile likelihoods. Parameter estimates for birth rates by initial cell number are *b*_2_ = 0.00793 [0.00785−0.00794], *b*_4_ = 0.00945 [0.0093−0.0096], and *b*_10_ = 0.0113 [0.0112−0.0114], and for death rates, the parameter estimates are *d*_2_ = 6.67 × 10^−18^ [−0.0005 to 0.0001], *d*_4_ = 0.0011 [0.0008−0.0013], and *d*_10_ = 0.00286 [0.0025−0.0028] for N_0_ = 2, 4, and 10, respectively. The data and code used to generate this figure can be found at https://github.com/brocklab/Johnson-AlleeGrowthModel.git.

To determine whether birth and/or death rates depend on the initial cell number, we first fit the data for initial cell number of N_0_ = 2, 4, and 10, grouped by initial condition N_0_ individually, to the stochastic simple birth and death model (Eqs 9 and 10) using the workflow described in Fig 3. The results of the fitting to the mean and variance in time to the simple birth–death model for each initial condition are shown in Fig 7 (7A, 7B and 7C for the mean and 7D, 7E and 7F for the variance). Each data set of a single initial condition N_0_ revealed identifiable birth and death rate parameters via profile likelihood analysis (see S13 Fig). Birth and death rate maximum likelihood parameter estimates are shown in Fig 7G, with confidence intervals obtained from the profile likelihood analysis (S13 Fig). Parameter estimates for birth rates by initial cell number are *b*_2_ = 0.00793 [0.00785–0.00794], *b*_4_ = 0.00945 [0.0093–0.0096], and *b*_10_ = 0.0113 [0.0112–0.0114], and for death rates, the parameter estimates are *d*_2_ = 6.67 × 10^−18^ [−0.0005 to 0.0001], *d*_4_ = 0.0011 [0.0008–0.0013], and *d*_10_ = 0.00286 [0.0025–0.0028] for N_0_ = 2, 4, and 10, respectively. The trend suggests a slight increase in net growth rate (birth rate minus death rate) with initial cell number, as is consistent with the preliminary growth rate analysis by initial cell number (Fig 6F) but inconsistent with the conventional exponential growth hypothesis, which should yield the same growth rate (and same birth and death rates), independent of the initial cell number.

### Fit of low seeding density data to all stochastic models reveals a weak Allee effect

**Fig 8:**
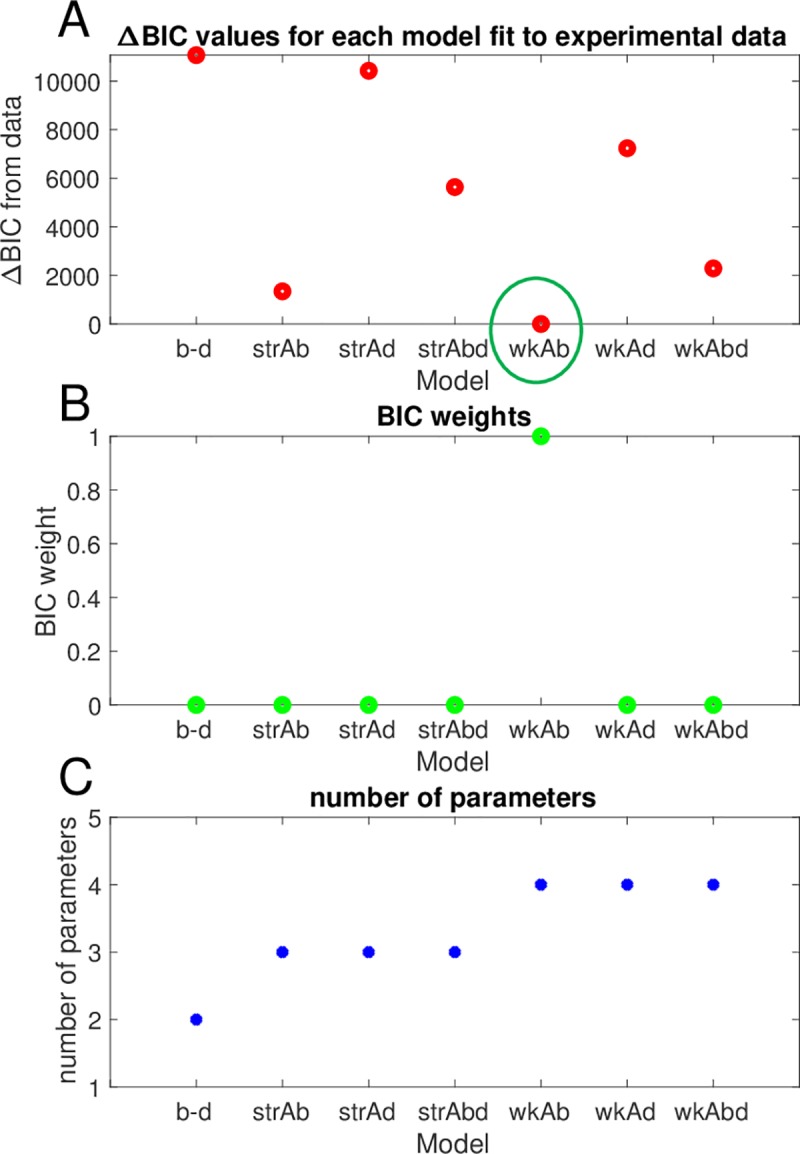
Weak Allee model on birth best describes BT-474 in vitro growth data. (A) *Δ*BIC values for the fit of the data to each of the 7 candidate stochastic growth models shows that the weak Allee model on birth exhibits the lowest BIC value. (B) BIC weights for each model indicate that the weak Allee model on birth is significantly better than all other models. (C) Number of parameters in each model as a measure of model complexity. The data and code used to generate this figure can be found at https://github.com/brocklab/Johnson-AlleeGrowthModel.git. BIC, Bayesian Information Criterion.

**Fig 9:**
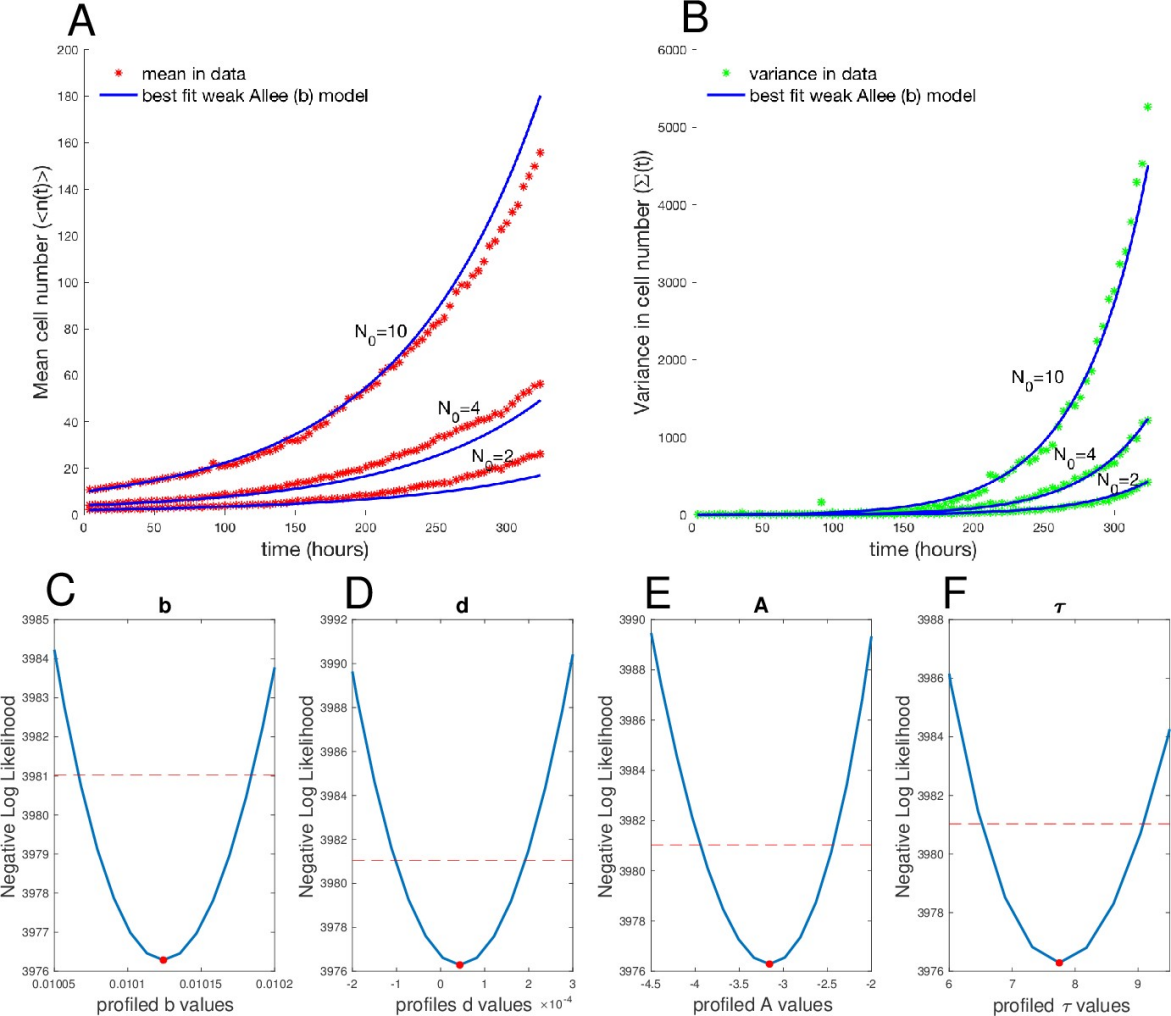
Mean and variance of data fit to a weak Allee model on birth. (A) Best fit of data mean to model mean displays the model fits the data well over all 3 initial conditions and over the time course. (B) Best fit of data variance to model variance displays the model fits the data well. (C) Profile likelihood analysis of birth rate around maximum likelihood *b* = 0.0101 [0.010068−0.010181]. (D) Profile likelihood analysis of death rate around maximum likelihood *d* = 4.3613 × 10^−5^ [−7.270 × 10^−5^ to 1.599 × 10^−4^]. (E) Profile likelihood analysis of Allee threshold *A* = −3.1576 [−3.8593 to − 2.4559]. (F) Profile likelihood analysis of the overall shape parameter *τ* = 7.480 [6.8871−9.0393]. The data and code used to generate this figure can be found at https://github.com/brocklab/Johnson-AlleeGrowthModel.git.

The growth data from the initial conditions of N_0_ = 2, 4, and 10 were combined and fit to each of the 7 candidate models using the moment-closure approximation workflow described in Fig 3 [42]. The BIC values for each model fit were computed and compared with the minimum BIC value (Fig 8A), and the corresponding BIC weights were calculated (Fig 8B) based on the goodness of fit and the complexity of the model (number of parameters; Fig 8C). We note that both the strong and weak Allee effect on birth models have significantly lower BIC values than the null model of the simple birth–death model (Fig 8A), providing strong, consistent evidence for the presence of an Allee effect in some form in this data set. Using the BIC weights to evaluate statistical significance between the models revealed that the weak Allee effect on birth is more likely than the strong Allee effect on birth model, with a BIC weight of essentially 1 to 0 for the weak Allee effect on birth versus the strong Allee effect on birth model. The best fit of the weak Allee effect on birth model to the mean and variance of the data is shown in Fig 9A and 9B, respectively (See S14–S19 Figs for the fit of the data to all 7 candidate models).

The profile likelihoods used to determine the 95% confidence intervals of the best fitting parameters of *b* = 0.0101 [0.010068–0.010181], *d* = 4.3613 × 10^−5^ [−7.270 × 10^−5^ to 1.599 × 10^−4^], *A* = −3.1576 [−3.8593 to −2.4559], and *τ* = 7.480 [6.8871–9.0393] are displayed in Fig 9C, 9D, 9E and 9F respectively. The discrepancy between the goodness of fit in the model mean and variance compared to the data is likely because an unbiased approach (as is prescribed in [42]) was used to weight the fit of both the mean and variance equally, using the likelihood function described in Eq 24. In theory, the relative weighting of the value of these 2 outputs could be tuned to reduce the error between the model and measurements in either the mean or variance. The results of model selection for the weak Allee effect model for the BT-474 data indicates that, outside of the effects of demographic stochasticity, any initial cell number is predicted to, on average, develop into a growing cell population, but the growth rate is expected to be significantly slower at low cell numbers.

## Discussion

The availability of single-cell resolution live imaging of cancer cell growth in a controlled in vitro setting starting at the population size of a single cell allowed us to examine in detail the influence of absolute cell number in a cell population on growth rate. Using mathematical modeling, we investigated the departure from simple first-order exponential growth kinetics in which the growth rate is proportional to the population size (cell number). Cell–cell interactions, as best known from quorum sensing in bacteria [55], underlie the cell-number dependence of growth rates. Most work on such dependence have been concerned with the slowing of growth with increasing cell number, e.g., due to approaching the carrying capacity of the cell culture. Here, we focus on the initiation of cell growth from a few individual cells and ask whether cooperative behavior or the Allee effect, as it is known from ecology, can be detected in a departure from exponential growth kinetics as predicted by mathematical models that consider the Allee effect. Because at the early stages of growth (from one cell or a few) growth kinetics is subjected to stochastic fluctuations due to small cell numbers, we formulated stochastic models that consider the Allee effect. We have demonstrated a framework for testing the relevance of a set of stochastic models of cancer cell growth applied to high-throughput, single-cell resolution data.

The 7 distinct candidate stochastic models of growth describe various modifications of the exponential growth model by incorporating growth-rate dependencies on the size of the population. The average behaviors of these models are examined in the deterministic form, and corresponding stochastic models that lead to the average behavior are developed. To test the relevance of the proposed stochastic models, the moment-closure approximation method [42] for parameter estimation in stochastic models (Fig 3) is applied to the high-throughput cell growth data. We first validated our framework by computational simulation of growth trajectories using a model of intermediate complexity. The parameter estimation framework was applied to the simulated data, confirming the ability of the framework to properly identify the underlying model structure and the true parameters. The framework is applied to a data set with a number of replicates from 3 initial conditions of N_0_ = 2, 4, and 10 BT-474 breast cancer cells. The fit of this growth data reveals that the weak Allee model with decreasing birth probability at a low cell number best describes the observed in vitro growth dynamics.

The presence of an Allee effect, even in the nutrient- and space-rich cell culture setting, implies that cancer cells likely exhibit cooperative growth. The ubiquitous cellular heterogeneity in tumors suggests that cooperative interactions between distinct subsets of cells must be present in order to maintain the observed heterogeneity [5, 56]. Evidence for noncell autonomous growth via eco-evolutionary interactions was recently observed by Kaznatcheev and colleagues [15], from which they observed a fitness benefit to combining fluorescently labeled cancer associated fibroblasts from parental and resistant cell lines and observed a benefit in growth rate of each independent cell type. Other microscopic experimental systems in which frequency dependent fitness effects have been considered include *Escherichia coli*, yeast, and other cancer cell types [15]. Recent work by Marusyk and colleagues [6] has found evidence for noncell autonomous proliferation using a mathematical modeling framework, showing that the null hypothesis of no clonal interactions can be easily rejected in favor of a model that considers a specific clone that helps support the growth of all other clones. Additionally, studies in which clonal diversity has been manipulated by combining clones in culture have demonstrated that the presence of diverse clones is necessary to obtain the observed growth rate achieved in multiclonal parental cell cultures [57].

Single-cell and clonal analysis has enabled the detection of secreted growth inducing factors, such as ILII [6], Wnt1 [5], IGFIII [7], and other paracrine factors [8, 58] in certain clones that result in an increased growth rate in the surrounding nonproducing clones. Bioinformatic analysis of single-cell gene expression data has allowed for the identification of specific subsets of cells that produce high levels of certain ligands and coexist in a population with cells that contain high expression levels of the cognate receptors [9, 59, 60]. Prior to single-cell analysis capabilities, these types of interactions were not readily detectable from bulk gene expression measurements. In such data, the coexpression of a ligand and its cognate receptor in the same sample (a cell population) has by default been interpreted as autocrine signaling [60]. Both paracrine and autocrine signaling are likely to play a significant and varying role in tumor growth.

In the field of tumor growth modeling, a few studies have considered the role of the Allee effect and the importance of incorporating it to describe and predict the effects of cooperative growth. Bottger and colleagues [38] developed a stochastic model in which an Allee effect naturally manifests based on assumptions that cancer cells can either exist in a migratory state or a proliferative state. Additional theoretical work has focused on spatial interactions between cancer cells and incorporated the Allee effect in a model for spatial spreading of cancer [37]. However, most classical tumor growth models rely on the assumption that early stage growth dynamics match the single exponential growth model [17, 25–27, 31, 61]. The weak Allee effect revealed in this work provides evidence that descriptions of early stage growth dynamics, which are relevant to progression, relapse, and metastasis, may be improved by taking into account the expected slowing of growth at low cell numbers. Beyond improving predictions of tumor growth and relapse dynamics, a model that considers the Allee effect may help to explain how cancer cell populations are able to go extinct after therapy despite the prediction of the log-kill hypothesis, which states that the probability of a cell being present after treatment, if a tumor is initially large, is greater than zero [62].

Although much work in tumor biology has led to an appreciation for cancer as an evolutionary process, a focus on cancer cells as ecosystems of interacting species or subpopulations may yield new insights. The possibility of exploiting ecology for the treatment of tumors based on studies in conservation biology about extinction and control of invasive species has been previously proposed [13, 14, 16–24]. However, this is the first work to our knowledge that has explicitly tested for the presence of the Allee effect in a regime in which low cancer cell populations can be measured and fit to a number of stochastic model structures representing different biological hypotheses about the Allee effect. Our finding is consistent with preclinical [2] and clinical observations [3, 63] of threshold-like behavior of tumor growth or slowed tumor growth following resection. Evidence for the Allee effect is also consistent with evidence of cooperation among cancer cell subclones as has been amply demonstrated [5–7, 64–66]. An understanding of subpopulation interactions and their molecular mediators that drive the observed Allee effect offer new approaches to manipulate cancer cell growth dynamics in favor of extinction. Allee effect models have been used to compare the impact of alternative management scenarios on threatened or exploited populations that are not readily accessible to experimentation [4]. Although the models themselves are phenomenological, the principles behind them, such as growth promoting cooperation, have been confirmed by ecological observations. The concept of cooperation promoting growth is intuitive to both the ecologist and modeler, in a similar fashion to the way we understand the carrying capacity term in the logistic growth model to represent the biological phenomena of slowing growth due to finite resources and space, and in the same manner, we intend knowledge of the Allee effect to be useful in a variety of contexts.

This study, which seeks to establish feasibility of detection and mathematical description of the Allee effect by observing growth kinetics, has obvious limitations with respect to biological interpretation of the relevance of results. Most notably, we apply the modeling and analysis framework to an in vitro data set for a single breast cancer cell line. The in vitro system may not faithfully represent in vivo growth dynamics, although we expect, and others have shown evidence that [3, 63], the Allee effect would only be more pronounced in vivo. An in vitro setting provides cells with all of the growth factors, nutrients, and space to robustly grow at low cell densities, whereas these factors may be less abundant for tumor cells in vivo at a low cell density. Although numbers of replicates for each initial condition N_0_ were relatively high (20 to 50 replicates) compared with typical growth studies, an increase in the number of replicates would likely lead to an improvement because the variance in the data should increase in accuracy with increasing sample size. In order to confirm that the Allee effect is a hallmark of tumor growth, a wide range of tumor types will need to be investigated. Additionally, the model presented here is phenomenological; we do not infer the mechanisms by which an Allee effect may be occurring such as in [37, 38], nor do we explicitly develop a model of subpopulation interactions as had been done in [6]. Future work will focus on investigating the molecular and cellular mechanisms for an Allee effect and developing a model of heterotypic subpopulation interactions that also considers phenotypic plasticity [67–70].

This work provides a framework for in-depth investigation of mathematical models of stochastic growth that incorporate the Allee effect and shows that an Allee effect model may be more suitable to describing early stage tumor growth dynamics than the exponential model. The potential role of the Allee effect opens a variety of new possibilities for understanding and controlling tumor growth. Biological mechanisms of cooperative growth that may be critical for cell populations to enter a highly proliferative regime need to be further investigated, because these mechanisms may be critical to preventing and predicting metastases and tumor relapse.

## Materials and methods

### Data processing and analysis

All mathematical modeling and analysis was performed in MATLAB. Code and data for all analysis is available on Github at: https://github.com/brocklab/Johnson-AlleeGrowthModel.git.

### Cell culture and low cell density seeding

The human breast cancer cell line BT-474 was used throughout this study. BT-474 is a standard cell line from ATCC. Cell lines were maintained and studied in Dulbecco’s Modified Eagle Medium (DMEM, Thermo Fischer Montreal, Canada) supplemented with insulin (Gibco Gaithersburg, MD) and 10% fetal bovine serum (Gibco) and 1% Penicillin-Streptomycin (Gibco Gaithersburg, MD Gaithersburg, MD). A subline of the BT-474 breast cancer cell line was engineered to constitutively express enhanced green fluorescent protein (EGFP) with a nuclear localization signal (NLS). Genomic integration of the EGFP expression cassette was accomplished through the Sleeping Beauty transposon system [71]. The EGFP-NLS sequence was obtained as a gBlock from IDT and cloned into the optimized Sleeping Beauty transfer vector psDBbi-Neo. pSBbie-Neo was a gift from Eric Kowarz (Addgene plasmid #60525) [71]. To mediate genomic integration, this two-plasmid system consisting of the transfer vector containing the EGFP-NLS expression cassette and the pCMV(CAT)T7-SB100 plasmid containing the Sleeping Beauty transposase was co-transfected into a BT-474 cell population using Lipofectamine 2000. mCMV(CAT)T7-SB100 was a gift from Zsuzsanna Izsvak (Addgene plasmid # 34879) [72]. GFP+ cells were collected by fluorescence activated cell sorting. BT-474-EGFPNLS1 cells are maintained in DMEM (Gibco Gaithersburg, MD) supplemented with insulin (Sigma Life Science St. Louis, MO), 10% fetal bovine serum (Fisher), and 200 μg/mL G418 (Caisson Labs Smithfield, UT). Cells were grown in precoated culture dishes at 37°C in a humidified, 5% CO_2_, 95 air atmosphere. Cells were seeded into the center 60 wells of a 96-well plate (Trueline Saint-Anne-de-Bellevue, Quebec, Canada) at precise initial cell numbers using fluorescence activated cell sorting (BD Fusion Franklin Lakes, NJ) plate sorting at single-cell precision. Plates were kept in the Incucyte Zoom, a combined incubator and time-lapsed microscope. Initial cell seeding numbers were verified by eye at 4× magnification using an image taken within 4 hours from the FACS seeding. Low cell density cultures were allowed to grow in media for 7 days and were subsequently fed fresh media every 2 to 3 days for up to 2 weeks.

### Time-lapse imaging

Time-lapse recordings of the cell cultures were performed using the whole-well imaging feature in the Incucyte Zoom (Essen Biosceince Ann Arbor, MI). Cells were maintained in the Incucyte at 37°C in humidified 5% CO_2_ atmosphere. Phase contrast and green-channel images were collected every 4 hours for up to 2 weeks.

### Image analysis

Recorded green-channel images were analyzed using the built-in analysis program in the Incucyte Zoom (Essen Bioscience Ann Arbor, MI) software analysis package. The true initial cell number of each well was confirmed by eye from the images at 4× magnification, and cell-number trajectories were binned accordingly. For each 96-well plate, an image processing definition was optimized using the built-in software and confirmed by eye to account for background fluorescence and local bubbles. Wells whose cells died off or did not exhibit any growth and wells without of focus images were removed from analysis.

## Supporting information

S1 Table Deterministic model structures to describe 3 distinct tumor growth dynamic model hypotheses. (PPTX)

S2 Table Equations to describe each of the stochastic model structures. (PPTX)

S1 Fig Illustrative example demonstrates that increasing magnitude of birth and death rate parameters increases variance in time, enabling the identifiability of *b* + *d*. The net growth (*b* − *d*) was held constant and the magnitude of *b* and *d* were simultaneously increased in order to demonstrate the effect on the time evolution of the variance. This example is used to explain intuitively how the measurement of variance in time enables the proper identification of the *b* and *d* parameters uniquely, even while the time evolution of the mean cell number remains constant. The data and code used to generate this figure can be found at https://github.com/brocklab/Johnson-AlleeGrowthModel.git.
(TIF)

S2 Fig Confirmation that moment approach derivations match measured mean and variance from simulated stochastic trajectories. (A) Example of stochastic growth model output from 5,000 simulated cell number trajectories starting at a single cell with birth rate of *b* = 0.0238 and a death rate of *d* = 0.005, revealing the expected variability in growth dynamics that is not averaged out at low initial numbers. (B) Stochastic growth trajectories uniformly samples every 4 hours. (C) Measured mean at each time interval from simulated data with model expected mean as a function of time for the true parameters overlaid. (D) Measured variance at each time interval from simulated data with model expected variance as a function of time for the true parameters overlaid. The data and code used to generate this figure can be found at https://github.com/brocklab/Johnson-AlleeGrowthModel.git.
(TIF)

S3 Fig Confirmation that moment approach derivations match measured mean and variance from simulated stochastic trajectories for strong Allee model on birth. (A) Measured mean at each time interval from simulated data with model expected mean as a function of time for the true parameters overlaid. (B) Measured variance at each time interval from simulated data with model expected variance as a function of time for the true parameters overlaid. The data and code used to generate this figure can be found at https://github.com/brocklab/Johnson-AlleeGrowthModel.git.
(TIF)

S4 Fig Confirmation that moment approach derivations match measured mean and variance from simulated stochastic trajectories for strong Allee model on death. (A) Measured mean at each time interval from simulated data with model expected mean as a function of time for the true parameters overlaid. (B) Measured variance at each time interval from simulated data with model expected variance as a function of time for the true parameters overlaid. The data and code used to generate this figure can be found at https://github.com/brocklab/Johnson-AlleeGrowthModel.git.
(TIF)

S5 Fig Confirmation that moment approach derivations match measured mean and variance from simulated stochastic trajectories for strong Allee model on birth and death. (A) Measured mean at each time interval from simulated data with model expected mean as a function of time for the true parameters overlaid. (B) Measured variance at each time interval from simulated data with model expected variance as a function of time for the true parameters overlaid. The data and code used to generate this figure can be found at https://github.com/brocklab/Johnson-AlleeGrowthModel.git.
(TIF)

S6 Fig Confirmation that moment approach derivations match measured mean and variance from simulated stochastic trajectories for weak Allee model on birth. (A) Measured mean at each time interval from simulated data with model expected mean as a function of time for the true parameters overlaid. (B) Measured variance at each time interval from simulated data with model expected variance as a function of time for the true parameters overlaid. The data and code used to generate this figure can be found at https://github.com/brocklab/Johnson-AlleeGrowthModel.git.
(TIF)

S7 Fig Confirmation that moment approach derivations match measured mean and variance from simulated stochastic trajectories for weak Allee model on death. (A) Measured mean at each time interval from simulated data with model expected mean as a function of time for the true parameters overlaid. (B) Measured variance at each time interval from simulated data with model expected variance as a function of time for the true parameters overlaid. The data and code used to generate this figure can be found at https://github.com/brocklab/Johnson-AlleeGrowthModel.git.
(TIF)

S8 Fig Confirmation that moment approach derivations match measured mean and variance from simulated stochastic trajectories for weak Allee model on birth and death. (A) Measured mean at each time interval from simulated data with model expected mean as a function of time for the true parameters overlaid. (B) Measured variance at each time interval from simulated data with model expected variance as a function of time for the true parameters overlaid. The data and code used to generate this figure can be found at https://github.com/brocklab/Johnson-AlleeGrowthModel.git.
(TIF)

S9 Fig Comparison of similar simple birth−death, strong, and weak Allee expectations for the time evolution of the mean and variance. (A) Expected time evolution of the mean cell number for the simple birth−death model (red), the strong Allee model on birth (blue), and the weak Allee model on birth (green) with the same birth and death rates for all but with *A* = 2 for the strong Allee model and *A* = −2, *τ* = 3 for the weak Allee model indicates significant differences in trajectories for *N*_0_ = 5, 10, and 15. (B) Expected time evolution of the variance in cell number for the same initial conditions and parameters. The data and code used to generate this figure can be found at https://github.com/brocklab/Johnson-AlleeGrowthModel.git.
(TIF)

S10 Fig Demonstration of effect of Allee mechanism on birth or death probability on the variance. (A) As expected, for constant parameters, the mean cell number in time for the strong model is the same for the strong Allee model on birth, death, or both. (B) The expected time evolution of the variance for the strong Allee model on the birth probability (cyan), death probability (dark blue), and both equally (black). (C) As expected, for constant parameters, the mean cell number in time for the weak Allee model is the same for the strong model on birth, death, or both. (D) The expected time evolution of the variance for the strong model on the birth probability (yellow), death probability (green), and both equally (black). The data and code used to generate this figure can be found at https://github.com/brocklab/Johnson-AlleeGrowthModel.git.
(TIF)

S11 Fig Decreased time resolution of data does not change fitting results; weak Allee model on birth is consistently chosen. (A) Example of down sampled time resolution from original data (red) to data every 24 hours (blue). (B) BIC values for each model fit at data sampled every 4, 12, and 24 hours, respectively, reveals weak Allee model has consistently the lowest BIC value and is chosen every time. The data and code used to generate this figure can be found at https://github.com/brocklab/Johnson-AlleeGrowthModel.git. BIC, Bayesian Information Criterion.
(TIF)

S12 Fig Normal cell culture density exhibits expected constant growth rate. (A) Thirty growth rate trajectories for seeding of *N*_0_ = 512 (green) and *N*_0_ = 1,024 (cyan). (B) Normalized cell number in time by *N*_0_ reveals expected constant growth rate. (C) Average growth rate and for *N*_0_ = 512 (green) of *g* = 0.0112 ± 0.00062 and *N*_0_ = 1,024 of *g* = 0.0115 ± 0.00074. The data and code used to generate this figure can be found at https://github.com/brocklab/Johnson-AlleeGrowthModel.git.
(TIF)

S13 Fig Profile likelihood analysis on birth and death rates for individual cell numbers *N*_0_ = 2, 4, and 10 reveals practical identifiability of the birth and death rate parameters for datasets of each individual group of *N*_0_ trajectories. (A, B, and C) Profile likelihood analysis on birth rate parameter for *N*_0_ = 2, 4, and 10, respectively. (D, E, and F) Profile likelihood analysis on death rate parameter for *N*_0_ = 2, 4, and 10, respectively. The data and code used to generate this figure can be found at https://github.com/brocklab/Johnson-AlleeGrowthModel.git.
(TIF)

S14 Fig Data fit to birth–death model results in a BIC = 1.9 × 10^4^. (A) Mean of the data (red) to the best fitting birth–death model mean (blue). (B) Variance of the data (green) to the best fitting birth-death model variance (blue). The data and code used to generate this figure can be found at https://github.com/brocklab/Johnson-AlleeGrowthModel.git. BIC, Bayesian Information Criterion.
(TIF)

S15 Fig Data fit to strong Allee on birth model results in a BIC = 9 × 10^3^. (A) Mean of the data (red) to the best fitting strong Allee on birth model mean (blue). (B) Variance of the data (green) to the best fitting strong Allee on birth model variance (blue). The data and code used to generate this figure can be found at https://github.com/brocklab/Johnson-AlleeGrowthModel.git. BIC, Bayesian Information Criterion.
(TIF)

S16 Fig Data fit to strong Allee on death model results in a BIC = 1.8 × 10^4^. (A) Mean of the data (red) to the best fitting strong Allee on death model mean (blue). (B) Variance of the data (green) to the best fitting strong Allee on death model variance (blue). The data and code used to generate this figure can be found at https://github.com/brocklab/Johnson-AlleeGrowthModel.git. BIC, Bayesian Information Criterion.
(TIF)

S17 Fig Data fit to strong Allee on birth and death model results in a BIC = 1.4 × 10^4^. (A) Mean of the data (red) to the best fitting strong Allee on birth and death model mean (blue). (B) Variance of the data (green) to the best fitting strong Allee on birth and death model variance (blue). The data and code used to generate this figure can be found at https://github.com/brocklab/Johnson-AlleeGrowthModel.git. BIC, Bayesian Information Criterion.
(TIF)

S18 Fig Data fit to weak Allee on death model results in a BIC = 1.5 × 10^4^. (A) Mean of the data (red) to the best fitting weak Allee on death model mean (blue). (B) Variance of the data (green) to the best fitting weak Allee on death model variance (blue). The data and code used to generate this figure can be found at https://github.com/brocklab/Johnson-AlleeGrowthModel.git. BIC, Bayesian Information Criterion.
(TIF)

S19 Fig Data fit to weak Allee on birth and death model results in a BIC = 1.0 × 10^4^. (A) Mean of the data (red) to the best fitting weak Allee on birth and death model mean (blue). (B) Variance of the data (green) to the best fitting weak Allee on birth and death model variance (blue). The data and code used to generate this figure can be found at https://github.com/brocklab/Johnson-AlleeGrowthModel.git. BIC, Bayesian Information Criterion.
(TIF)

S1 Text Stochastic model simulation using the Gillespie algorithm. (DOCX)

S2 Text Derivation of the moment-closure approximation for the first moment of the birth–death model. (DOCX)

S3 Text Confirmation that derivations of mean and variance for each model match the mean and variance from simulated data with known parameters. (DOCX)

S4 Text Theoretical identifiability of the structural models using the differential algebra approach applied to the simple birth–death model as an example. (DOCX)

S5 Text Model Selection using BIC and BIC weights. BIC, Bayesian Information Criterion.
(DOCX)

## Abbreviations

BIC: Bayesian Information Criterion
EGFP: enhanced green fluorescent protein
ME: master equation
NLS: nuclear localization signal
ODE: ordinary differential equation
