# Supplementary material for: Cancer cell population growth kinetics at low densities deviate from the exponential growth model and suggest an Allee effect
Source: PLoS Biol. 2019 Aug 5;17(8):e3000399. doi: 10.1371/journal.pbio.3000399 (PMC6695196; doi:10.1371/journal.pbio.3000399)
Supplement: S1 Table — (PPTX) [file pbio.3000399.s001.pptx]

## Slide 1
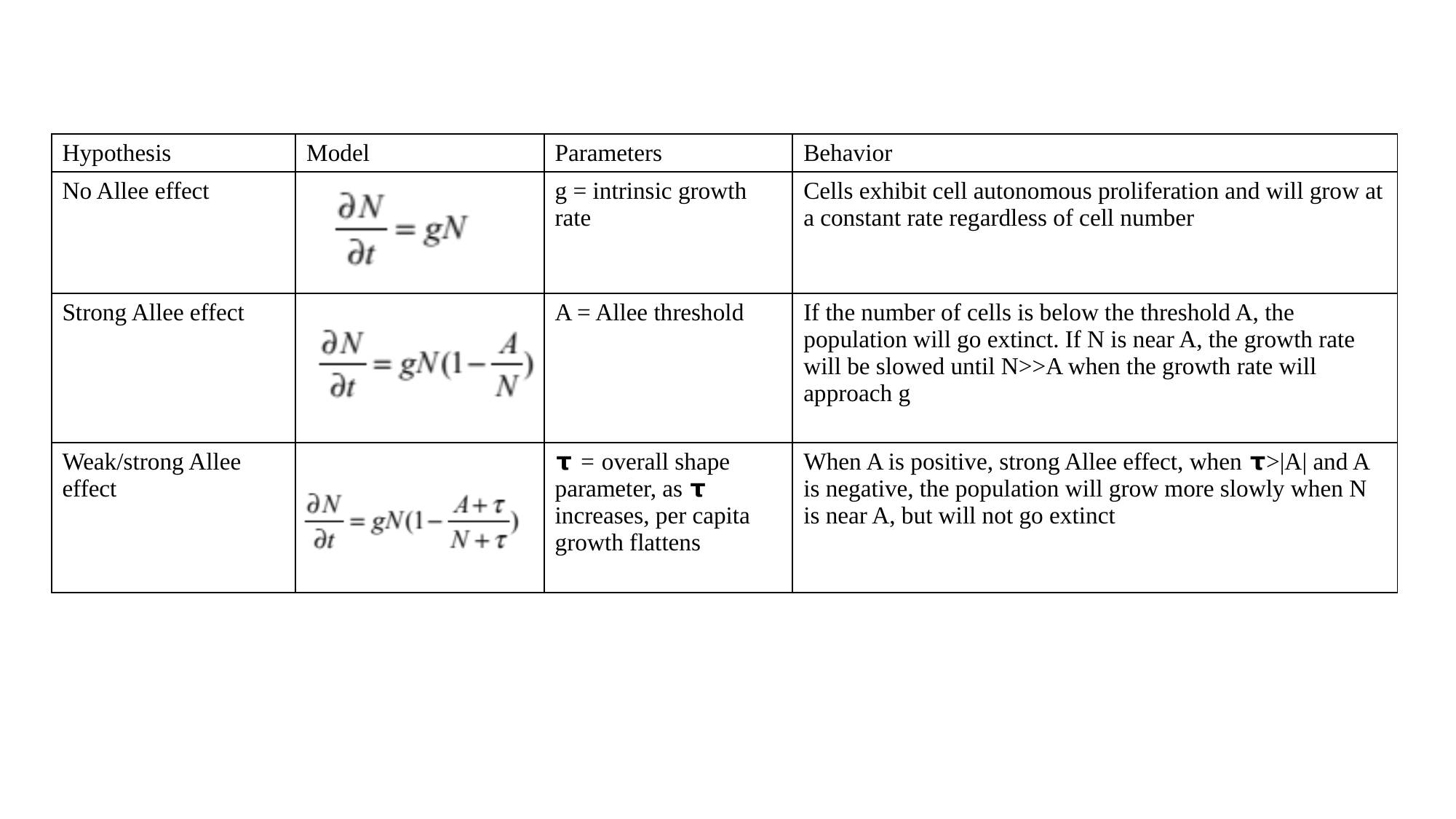

| Hypothesis | Model | Parameters | Behavior |
| --- | --- | --- | --- |
| No Allee effect | | g = intrinsic growth rate | Cells exhibit cell autonomous proliferation and will grow at a constant rate regardless of cell number |
| Strong Allee effect | | A = Allee threshold | If the number of cells is below the threshold A, the population will go extinct. If N is near A, the growth rate will be slowed until N>>A when the growth rate will approach g |
| Weak/strong Allee effect | | 𝞃 = overall shape parameter, as 𝞃 increases, per capita growth flattens | When A is positive, strong Allee effect, when 𝞃>|A| and A is negative, the population will grow more slowly when N is near A, but will not go extinct |
