# Supplementary material for: Cancer cell population growth kinetics at low densities deviate from the exponential growth model and suggest an Allee effect
Source: PLoS Biol. 2019 Aug 5;17(8):e3000399. doi: 10.1371/journal.pbio.3000399 (PMC6695196; doi:10.1371/journal.pbio.3000399)
Supplement: S2 Table — (PPTX) [file pbio.3000399.s002.pptx]

## Slide 1
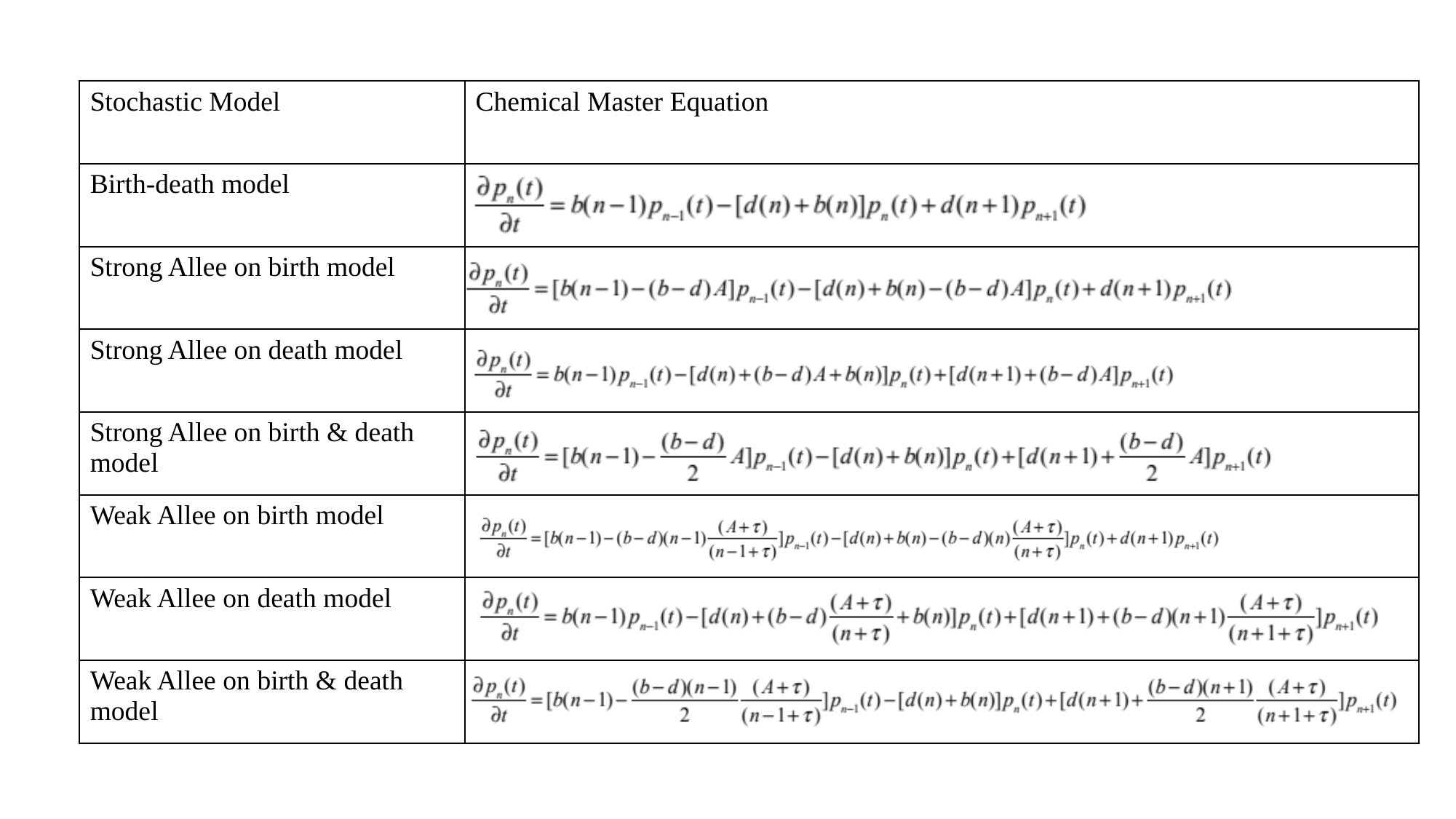

| Stochastic Model | Chemical Master Equation |
| --- | --- |
| Birth-death model | |
| Strong Allee on birth model | |
| Strong Allee on death model | |
| Strong Allee on birth & death model | |
| Weak Allee on birth model | |
| Weak Allee on death model | |
| Weak Allee on birth & death model | |
