# Supplementary figures and images for: Cancer cell population growth kinetics at low densities deviate from the exponential growth model and suggest an Allee effect
Source: PLoS Biol. 2019 Aug 5;17(8):e3000399. doi: 10.1371/journal.pbio.3000399 (PMC6695196; doi:10.1371/journal.pbio.3000399)

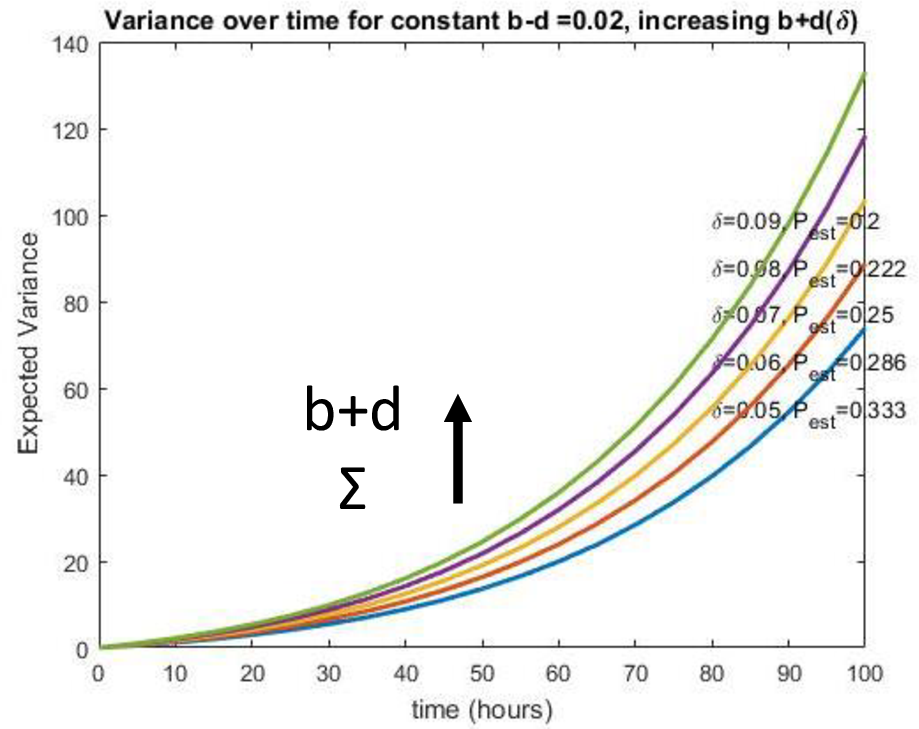

Supplement: S1 Fig — The net growth (b − d) was held constant and the magnitude of b and d were simultaneously increased in order to demonstrate the effect on the time evolution of the variance. This example is used to explain intuitively how the measurement of variance in time enables the proper identification of the b and d parameters uniquely, even while the time evolution of the mean cell number remains constant. The data and code used to generate this figure can be found at https://github.com/brocklab/Johnson-AlleeGrowthModel.git. (TIF) [file pbio.3000399.s003.tif]

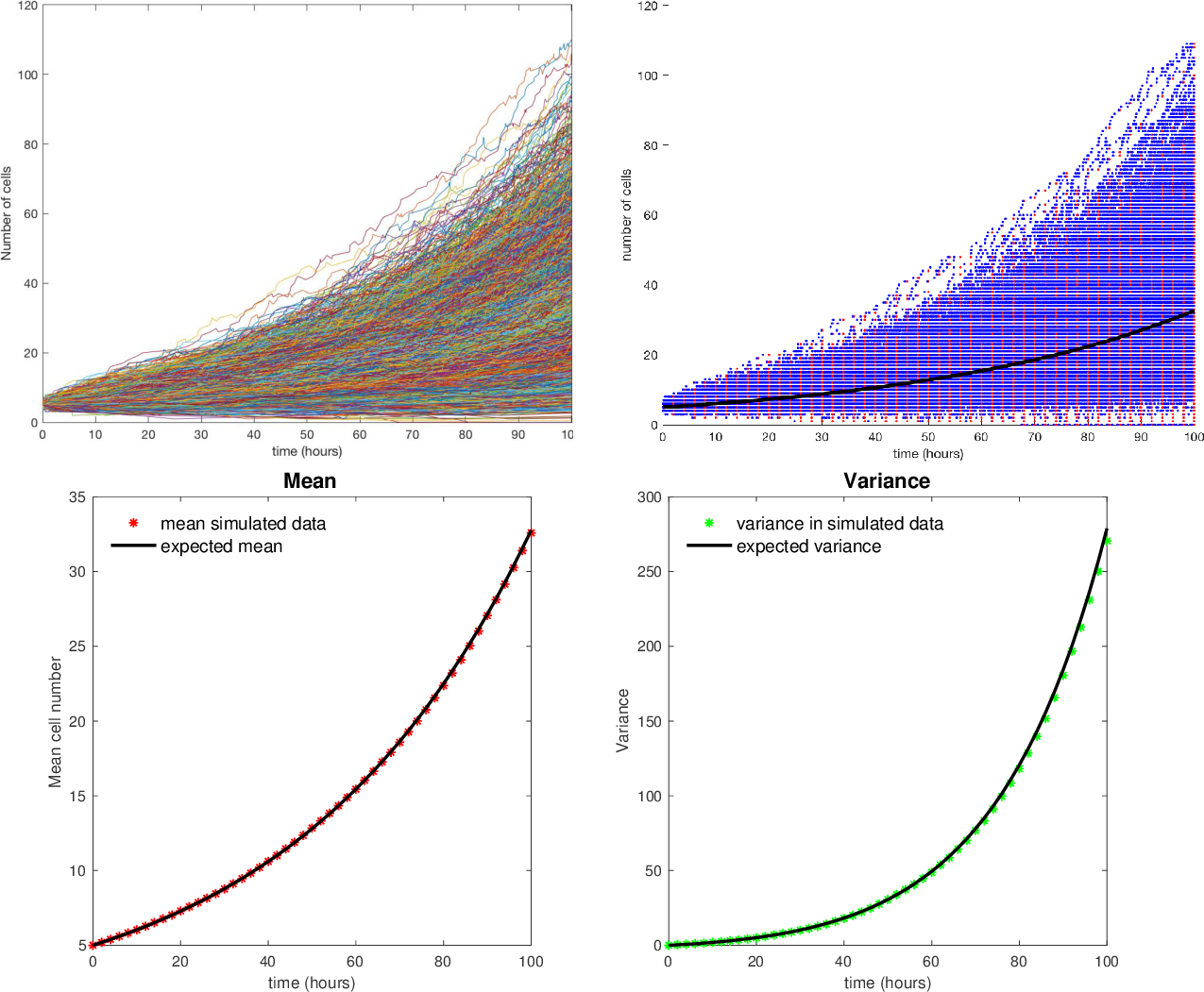

Supplement: S2 Fig — (A) Example of stochastic growth model output from 5,000 simulated cell number trajectories starting at a single cell with birth rate of b = 0.0238 and a death rate of d = 0.005, revealing the expected variability in growth dynamics that is not averaged out at low initial numbers. (B) Stochastic growth trajectories uniformly samples every 4 hours. (C) Measured mean at each time interval from simulated data with model expected mean as a function of time for the true parameters overlaid. (D) Measured variance at each time interval from simulated data with model expected variance as a function of time for the true parameters overlaid. The data and code used to generate this figure can be found at https://github.com/brocklab/Johnson-AlleeGrowthModel.git. (TIF) [file pbio.3000399.s004.tif]

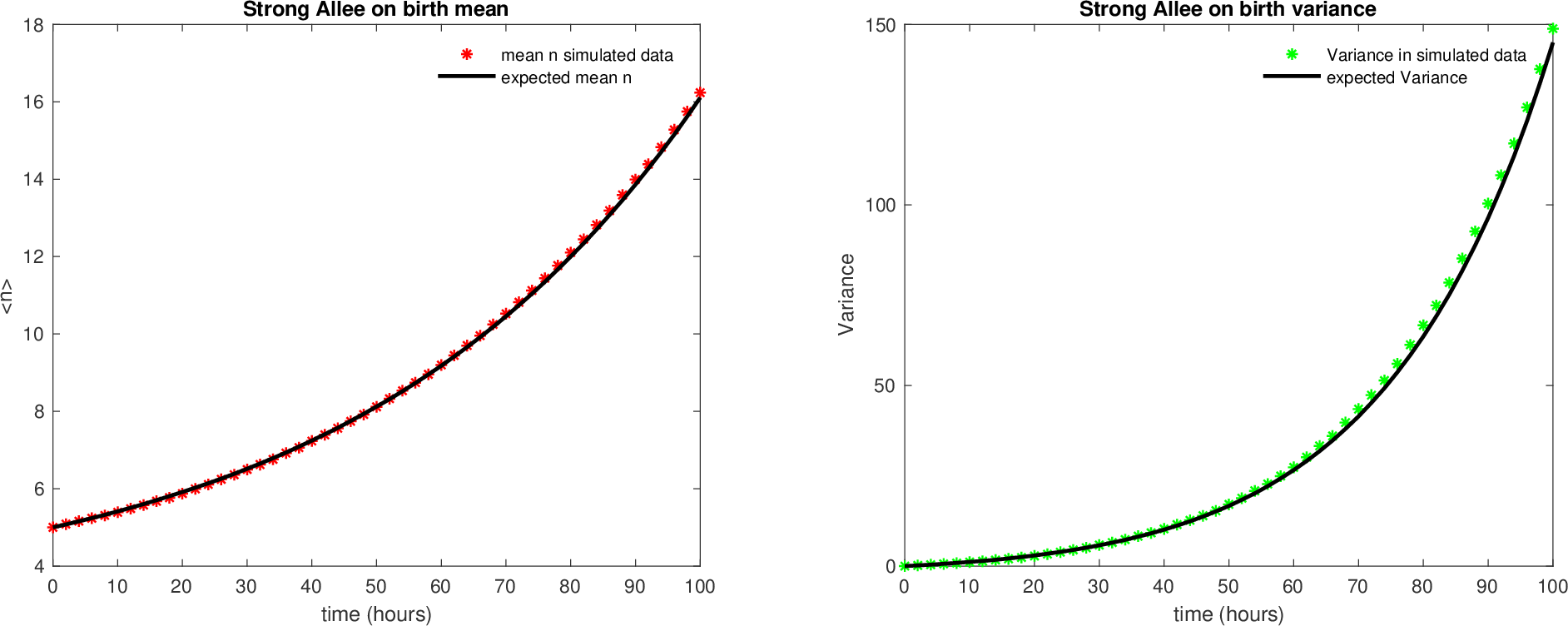

Supplement: S3 Fig — (A) Measured mean at each time interval from simulated data with model expected mean as a function of time for the true parameters overlaid. (B) Measured variance at each time interval from simulated data with model expected variance as a function of time for the true parameters overlaid. The data and code used to generate this figure can be found at https://github.com/brocklab/Johnson-AlleeGrowthModel.git. (TIF) [file pbio.3000399.s005.tif]

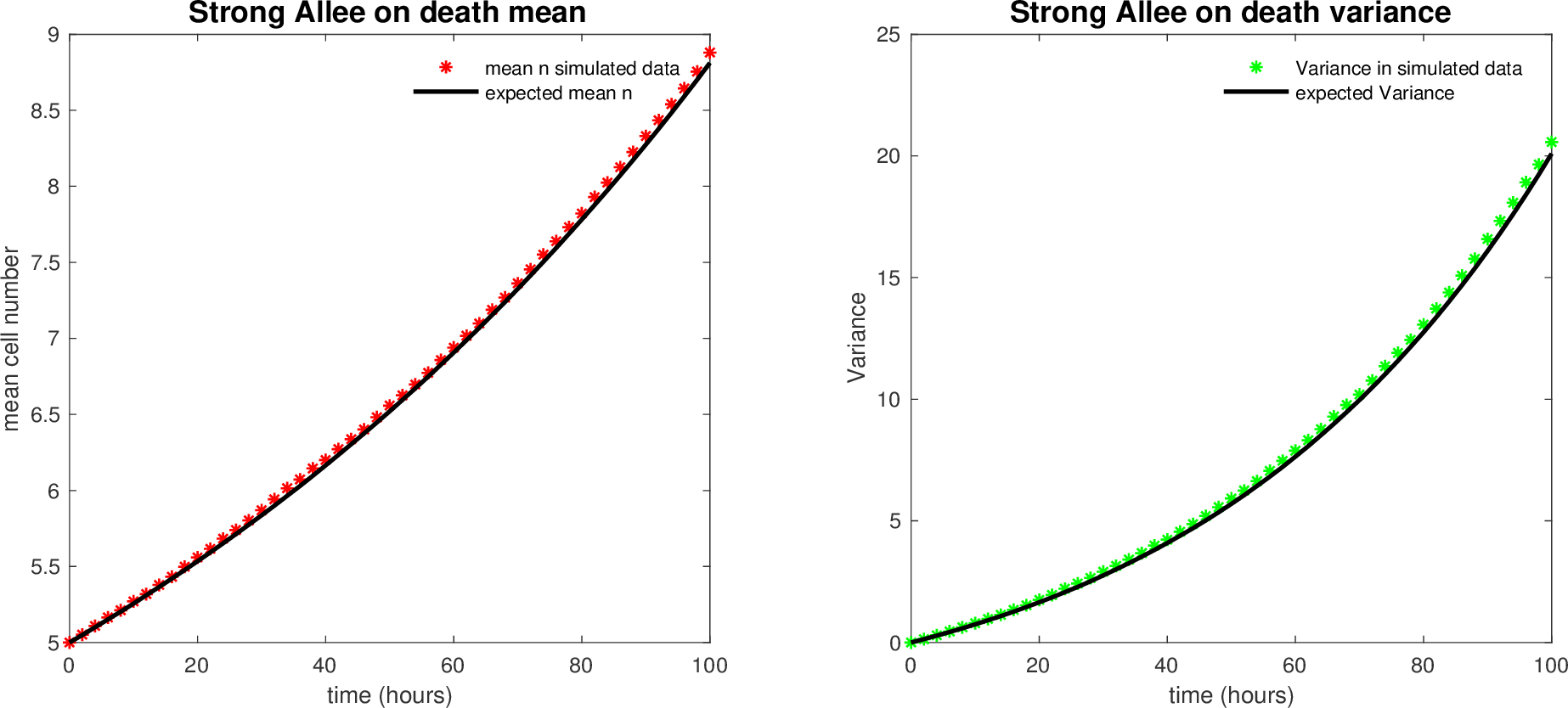

Supplement: S4 Fig — (A) Measured mean at each time interval from simulated data with model expected mean as a function of time for the true parameters overlaid. (B) Measured variance at each time interval from simulated data with model expected variance as a function of time for the true parameters overlaid. The data and code used to generate this figure can be found at https://github.com/brocklab/Johnson-AlleeGrowthModel.git. (TIF) [file pbio.3000399.s006.tif]

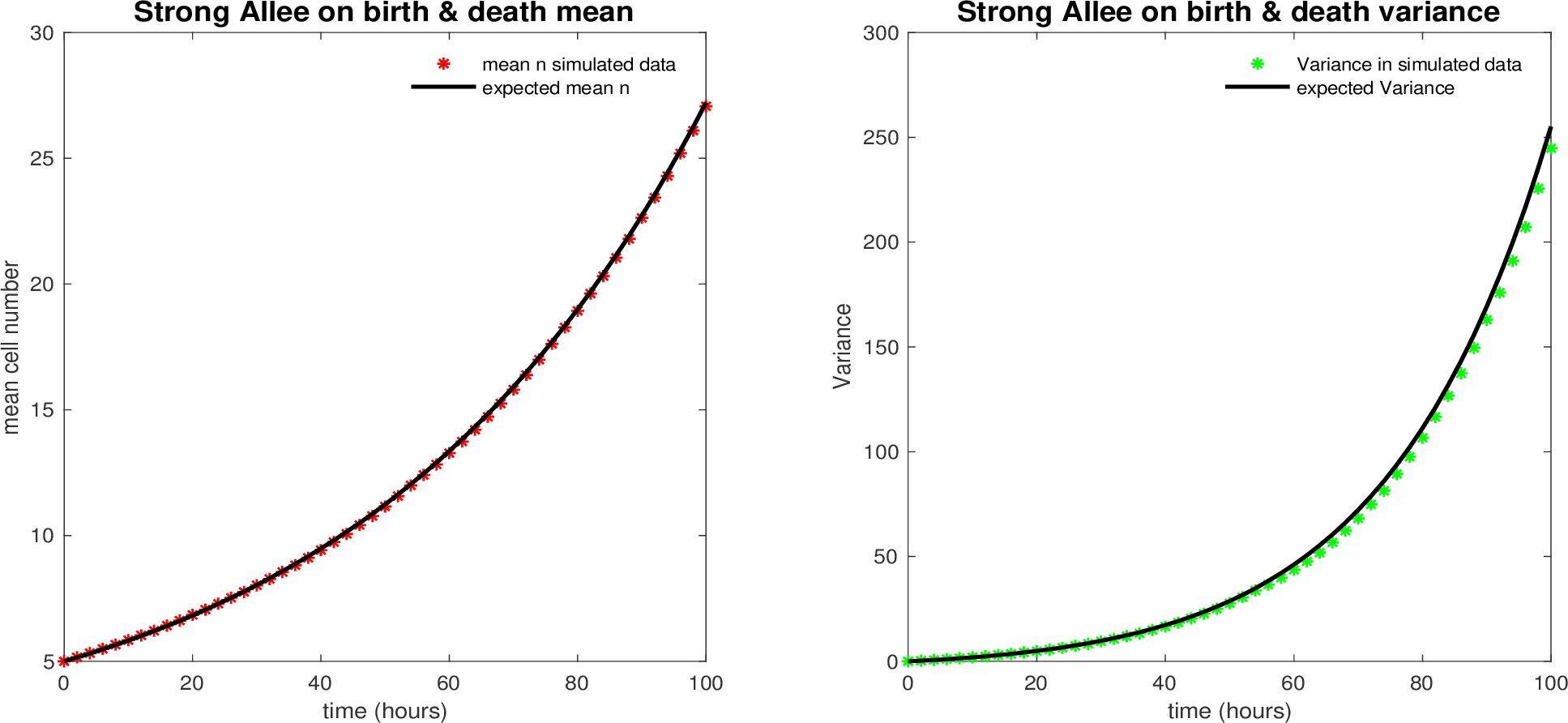

Supplement: S5 Fig — (A) Measured mean at each time interval from simulated data with model expected mean as a function of time for the true parameters overlaid. (B) Measured variance at each time interval from simulated data with model expected variance as a function of time for the true parameters overlaid. The data and code used to generate this figure can be found at https://github.com/brocklab/Johnson-AlleeGrowthModel.git. (TIF) [file pbio.3000399.s007.tif]

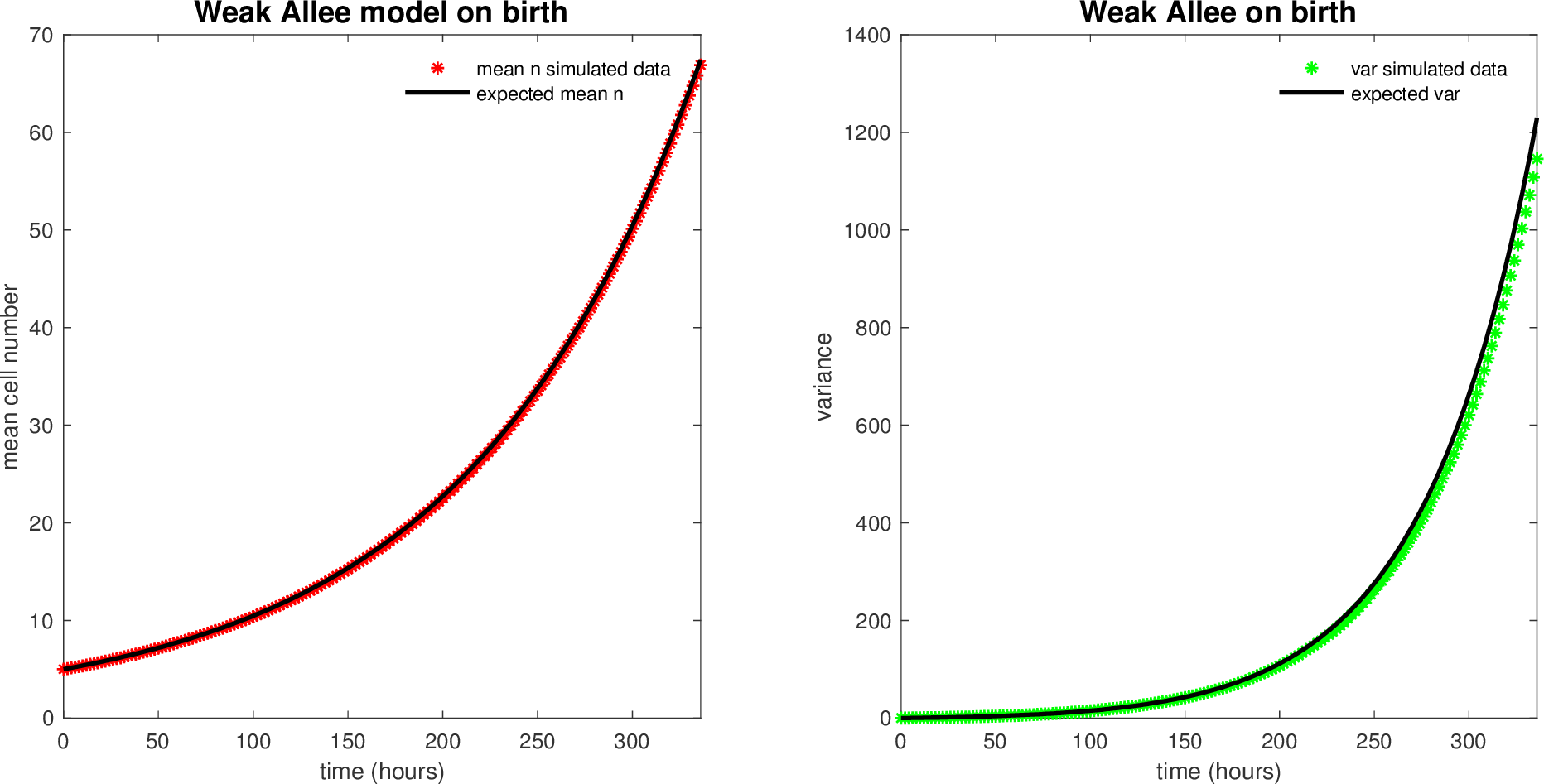

Supplement: S6 Fig — (A) Measured mean at each time interval from simulated data with model expected mean as a function of time for the true parameters overlaid. (B) Measured variance at each time interval from simulated data with model expected variance as a function of time for the true parameters overlaid. The data and code used to generate this figure can be found at https://github.com/brocklab/Johnson-AlleeGrowthModel.git. (TIF) [file pbio.3000399.s008.tif]

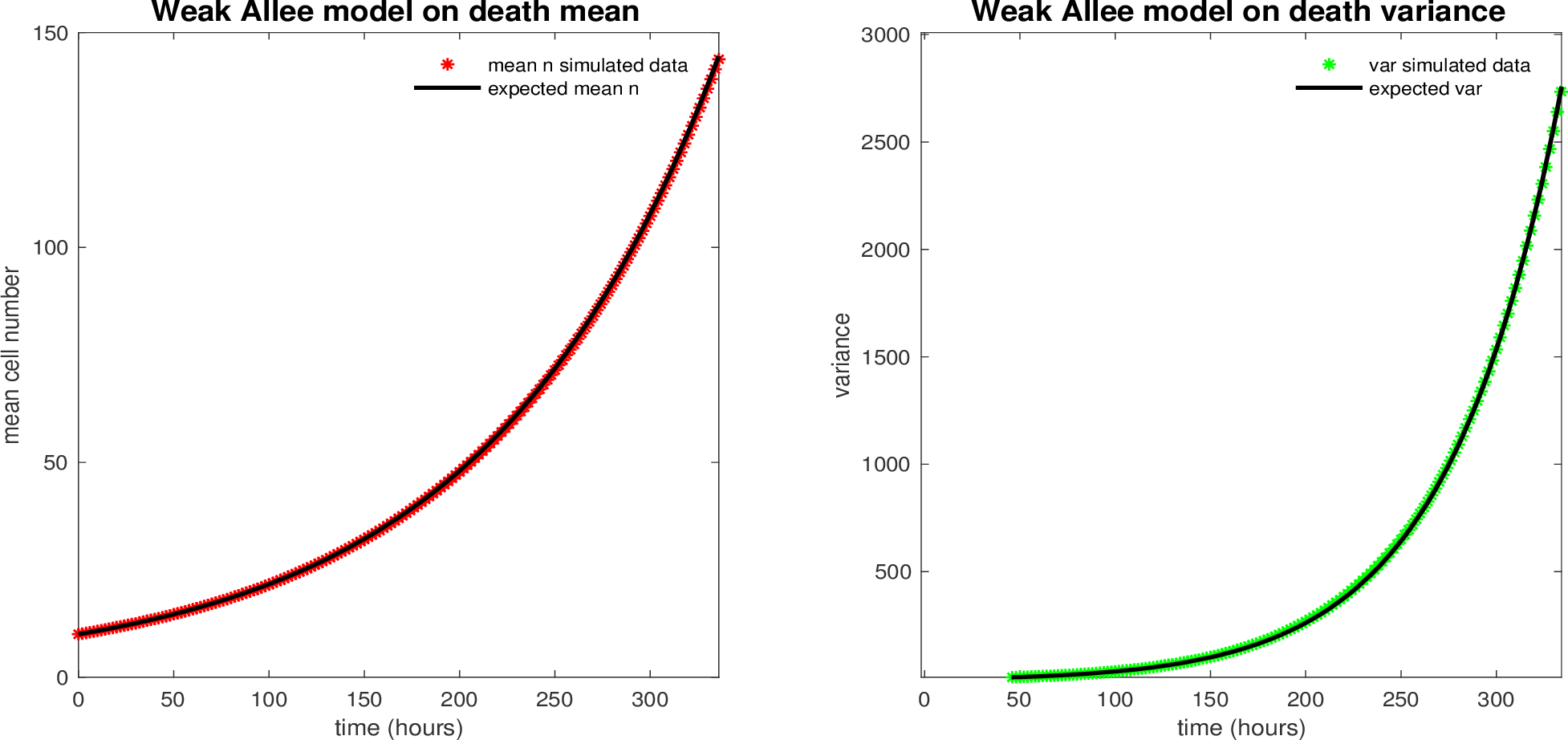

Supplement: S7 Fig — (A) Measured mean at each time interval from simulated data with model expected mean as a function of time for the true parameters overlaid. (B) Measured variance at each time interval from simulated data with model expected variance as a function of time for the true parameters overlaid. The data and code used to generate this figure can be found at https://github.com/brocklab/Johnson-AlleeGrowthModel.git. (TIF) [file pbio.3000399.s009.tif]

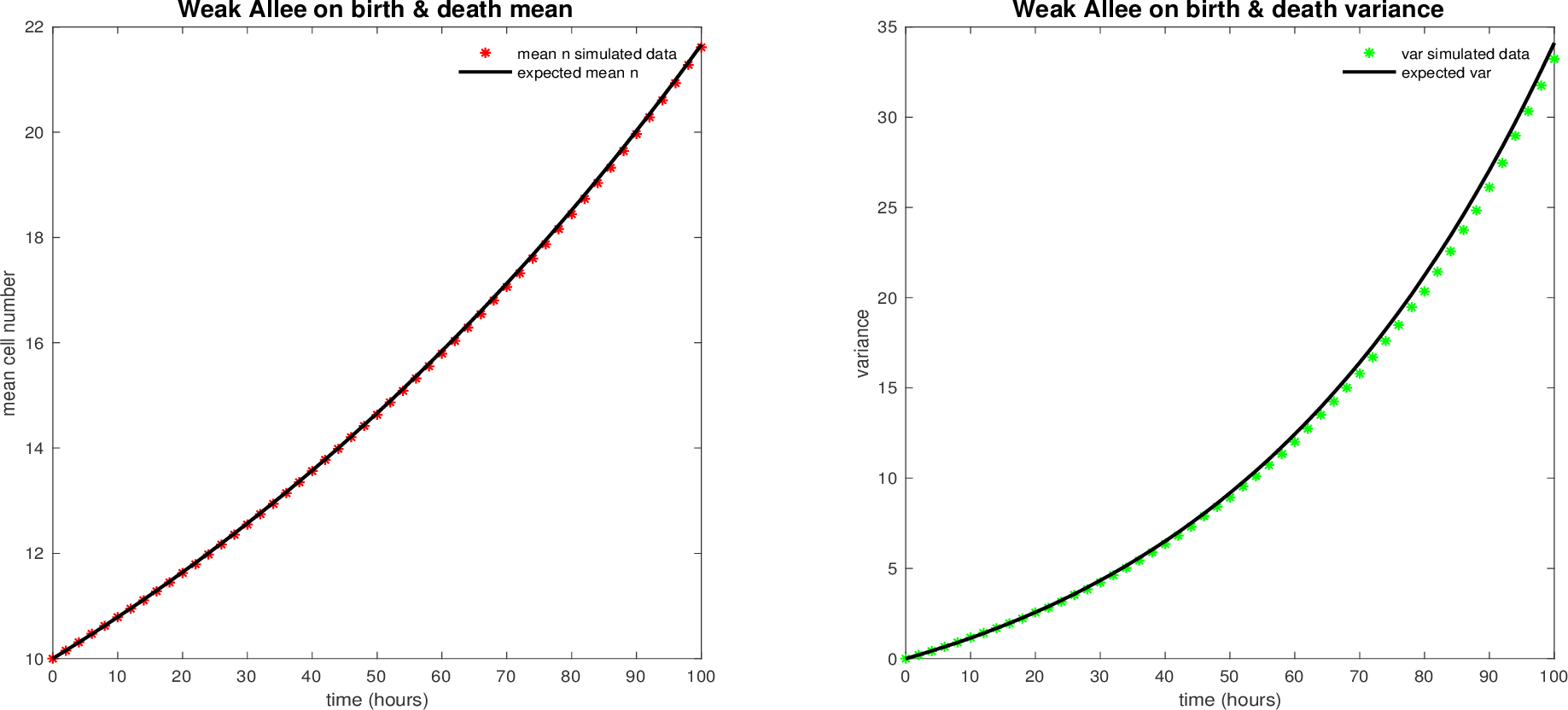

Supplement: S8 Fig — (A) Measured mean at each time interval from simulated data with model expected mean as a function of time for the true parameters overlaid. (B) Measured variance at each time interval from simulated data with model expected variance as a function of time for the true parameters overlaid. The data and code used to generate this figure can be found at https://github.com/brocklab/Johnson-AlleeGrowthModel.git. (TIF) [file pbio.3000399.s010.tif]

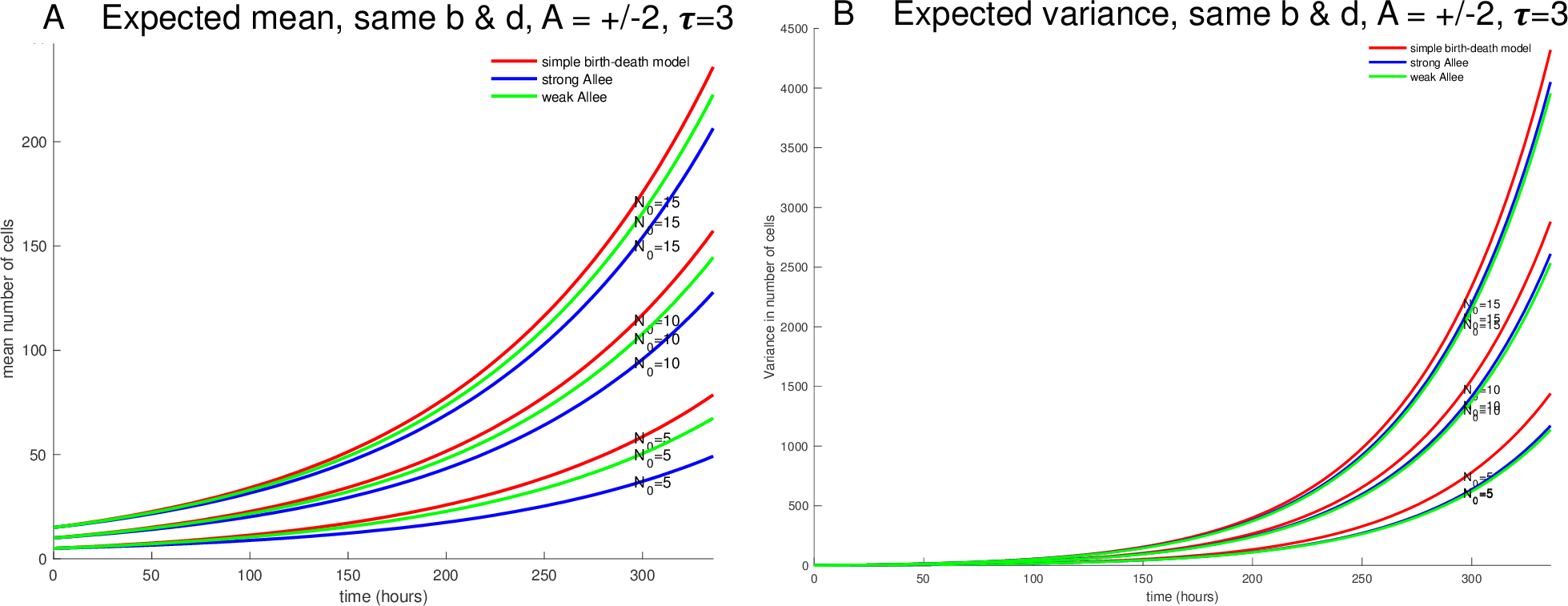

Supplement: S9 Fig — (A) Expected time evolution of the mean cell number for the simple birth−death model (red), the strong Allee model on birth (blue), and the weak Allee model on birth (green) with the same birth and death rates for all but with A = 2 for the strong Allee model and A = −2, τ = 3 for the weak Allee model indicates significant differences in trajectories for N0 = 5, 10, and 15. (B) Expected time evolution of the variance in cell number for the same initial conditions and parameters. The data and code used to generate this figure can be found at https://github.com/brocklab/Johnson-AlleeGrowthModel.git. (TIF) [file pbio.3000399.s011.tif]

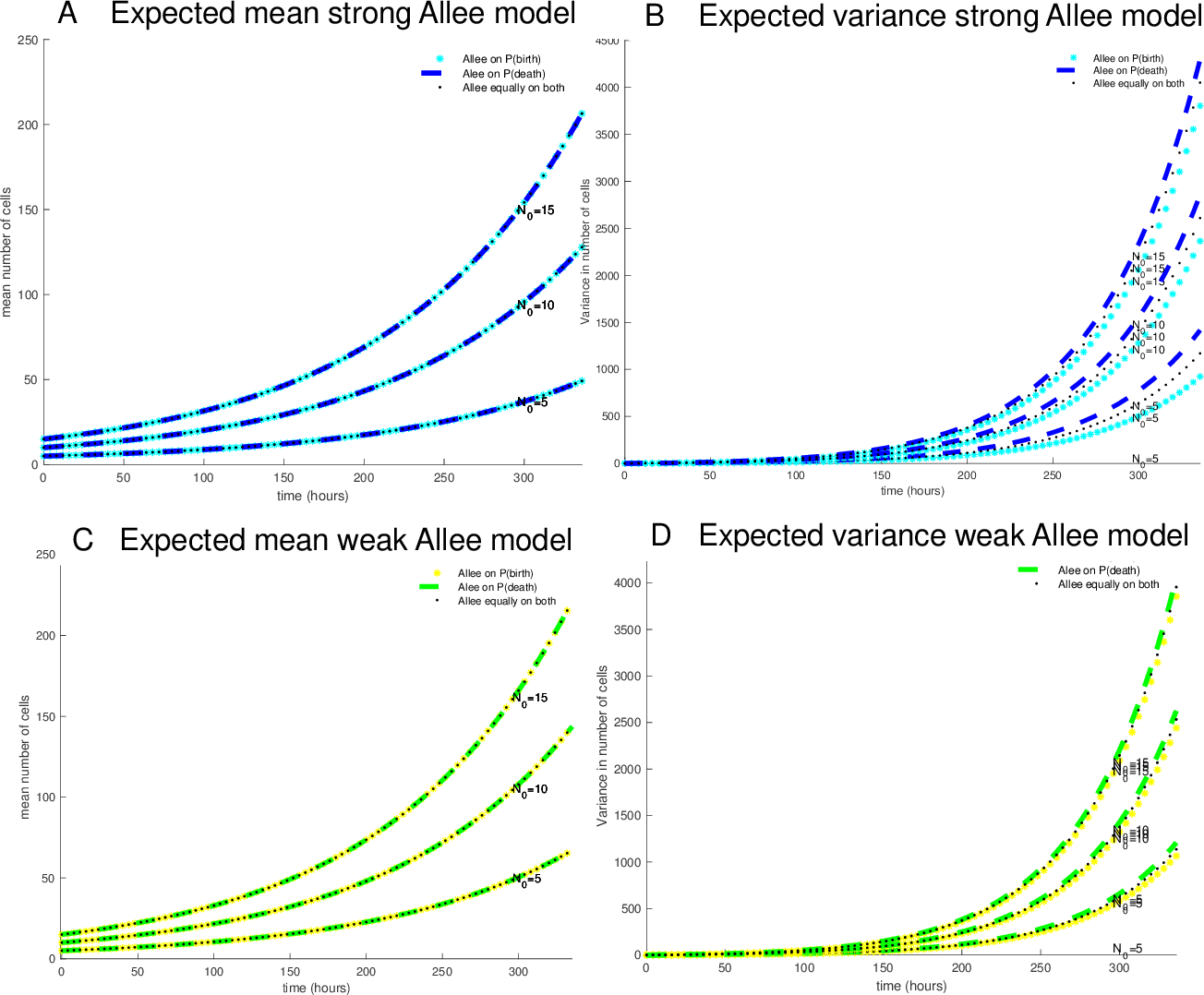

Supplement: S10 Fig — (A) As expected, for constant parameters, the mean cell number in time for the strong model is the same for the strong Allee model on birth, death, or both. (B) The expected time evolution of the variance for the strong Allee model on the birth probability (cyan), death probability (dark blue), and both equally (black). (C) As expected, for constant parameters, the mean cell number in time for the weak Allee model is the same for the strong model on birth, death, or both. (D) The expected time evolution of the variance for the strong model on the birth probability (yellow), death probability (green), and both equally (black). The data and code used to generate this figure can be found at https://github.com/brocklab/Johnson-AlleeGrowthModel.git. (TIF) [file pbio.3000399.s012.tif]

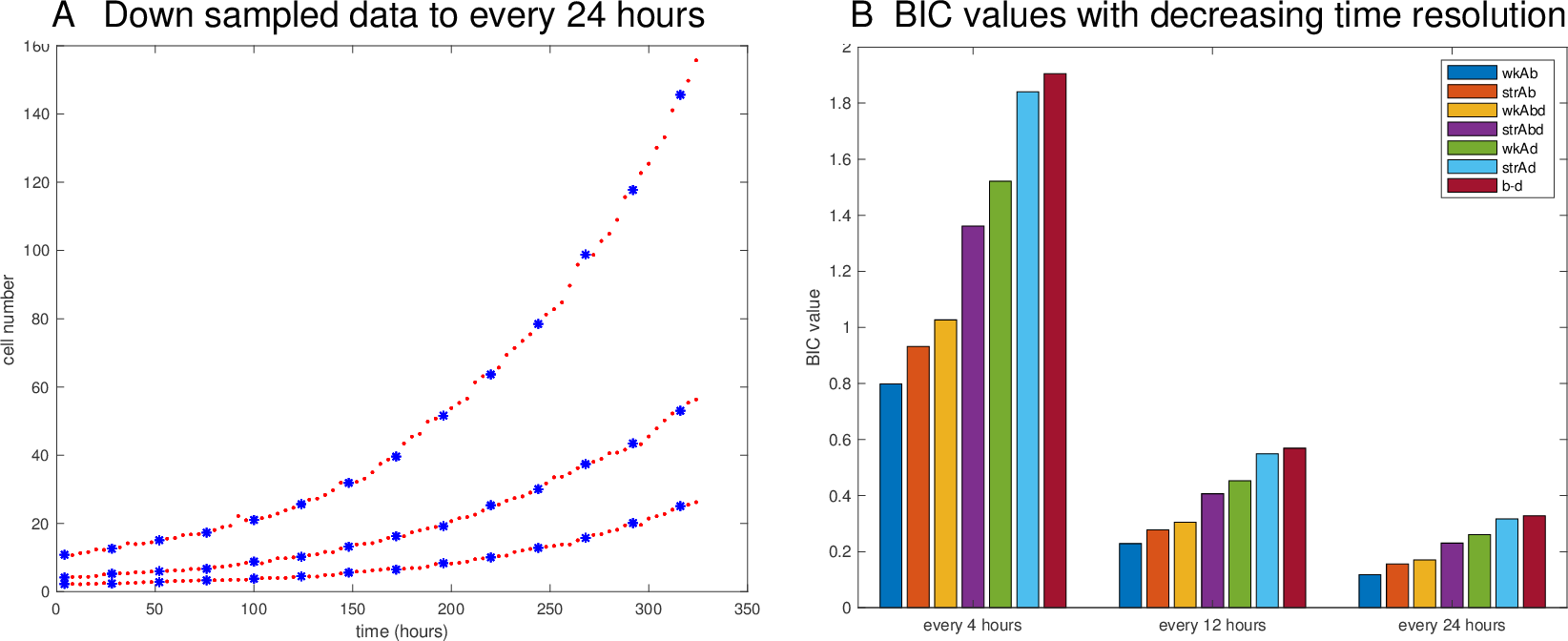

Supplement: S11 Fig — (A) Example of down sampled time resolution from original data (red) to data every 24 hours (blue). (B) BIC values for each model fit at data sampled every 4, 12, and 24 hours, respectively, reveals weak Allee model has consistently the lowest BIC value and is chosen every time. The data and code used to generate this figure can be found at https://github.com/brocklab/Johnson-AlleeGrowthModel.git. BIC, Bayesian Information Criterion. (TIF) [file pbio.3000399.s013.tif]

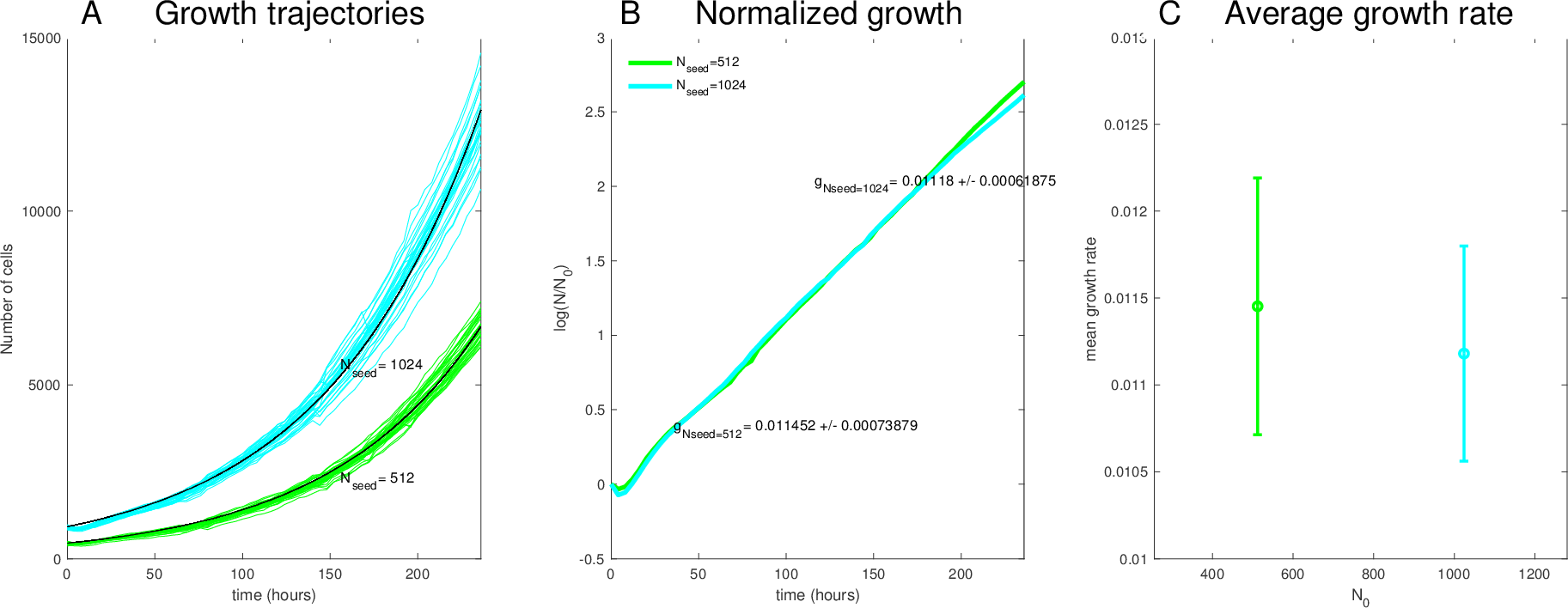

Supplement: S12 Fig — (A) Thirty growth rate trajectories for seeding of N0 = 512 (green) and N0 = 1,024 (cyan). (B) Normalized cell number in time by N0 reveals expected constant growth rate. (C) Average growth rate and for N0 = 512 (green) of g = 0.0112 ± 0.00062 and N0 = 1,024 of g = 0.0115 ± 0.00074. The data and code used to generate this figure can be found at https://github.com/brocklab/Johnson-AlleeGrowthModel.git. (TIF) [file pbio.3000399.s014.tif]

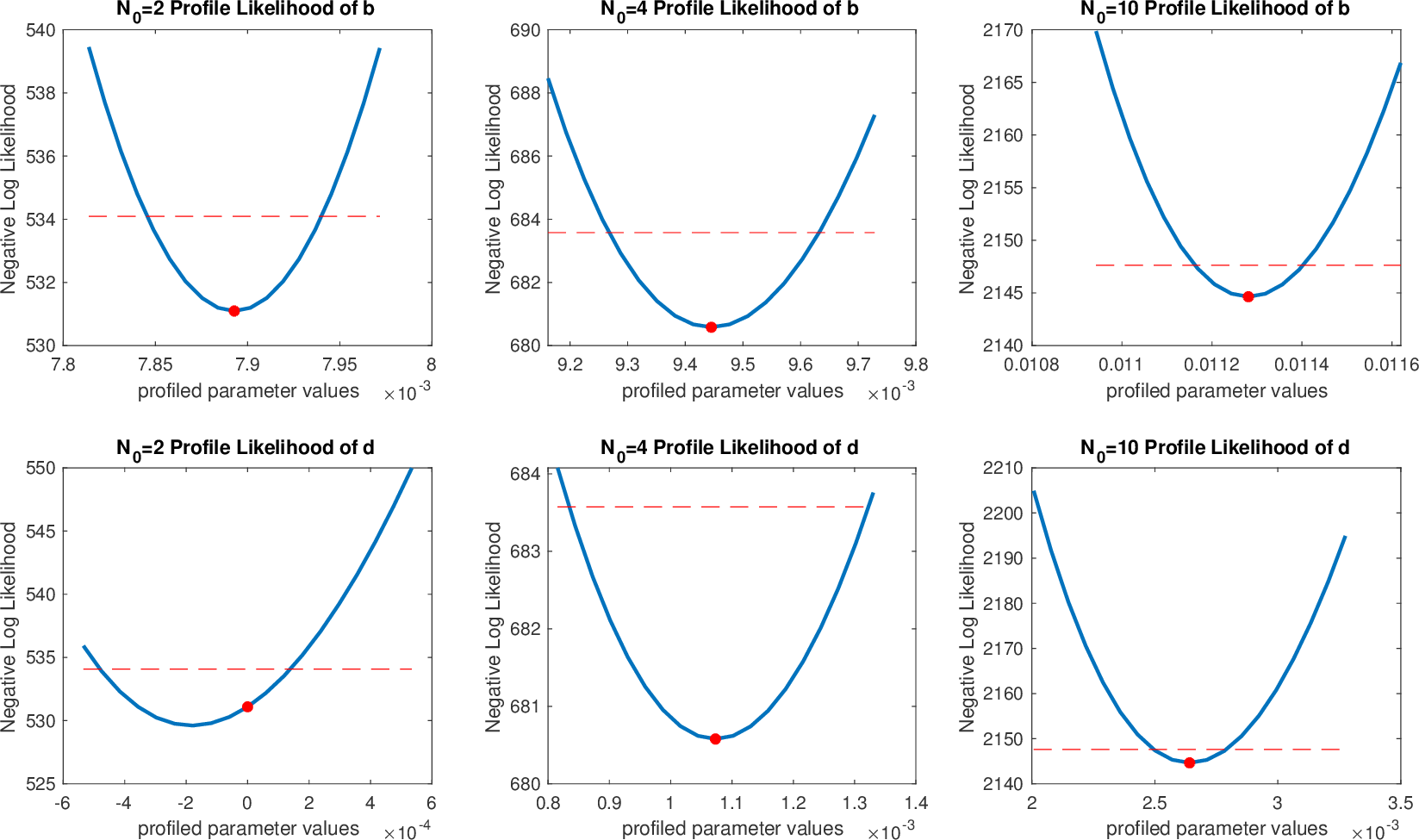

Supplement: S13 Fig — (A, B, and C) Profile likelihood analysis on birth rate parameter for N0 = 2, 4, and 10, respectively. (D, E, and F) Profile likelihood analysis on death rate parameter for N0 = 2, 4, and 10, respectively. The data and code used to generate this figure can be found at https://github.com/brocklab/Johnson-AlleeGrowthModel.git. (TIF) [file pbio.3000399.s015.tif]

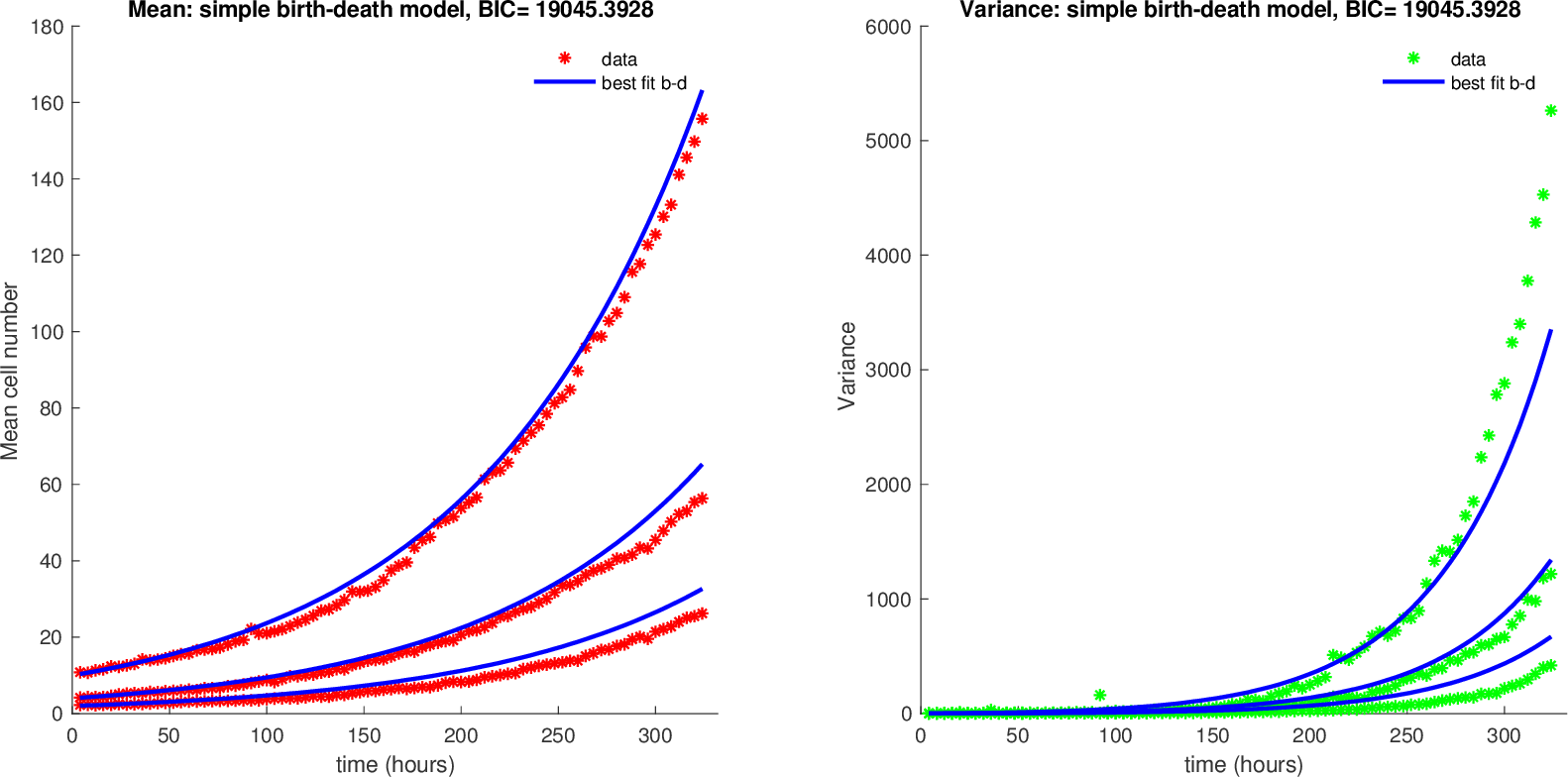

Supplement: S14 Fig — (A) Mean of the data (red) to the best fitting birth–death model mean (blue). (B) Variance of the data (green) to the best fitting birth-death model variance (blue). The data and code used to generate this figure can be found at https://github.com/brocklab/Johnson-AlleeGrowthModel.git. BIC, Bayesian Information Criterion. (TIF) [file pbio.3000399.s016.tif]

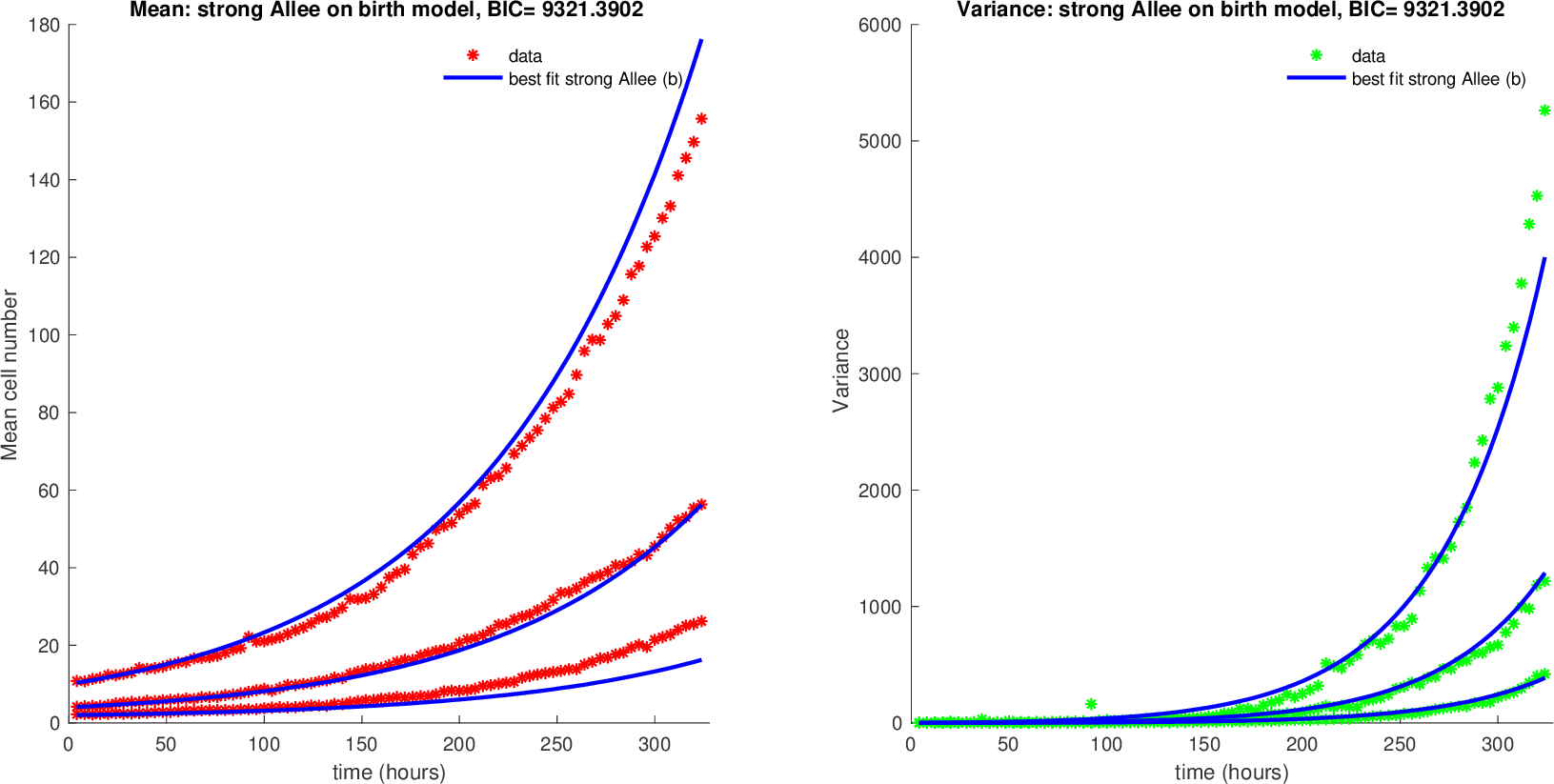

Supplement: S15 Fig — (A) Mean of the data (red) to the best fitting strong Allee on birth model mean (blue). (B) Variance of the data (green) to the best fitting strong Allee on birth model variance (blue). The data and code used to generate this figure can be found at https://github.com/brocklab/Johnson-AlleeGrowthModel.git. BIC, Bayesian Information Criterion. (TIF) [file pbio.3000399.s017.tif]

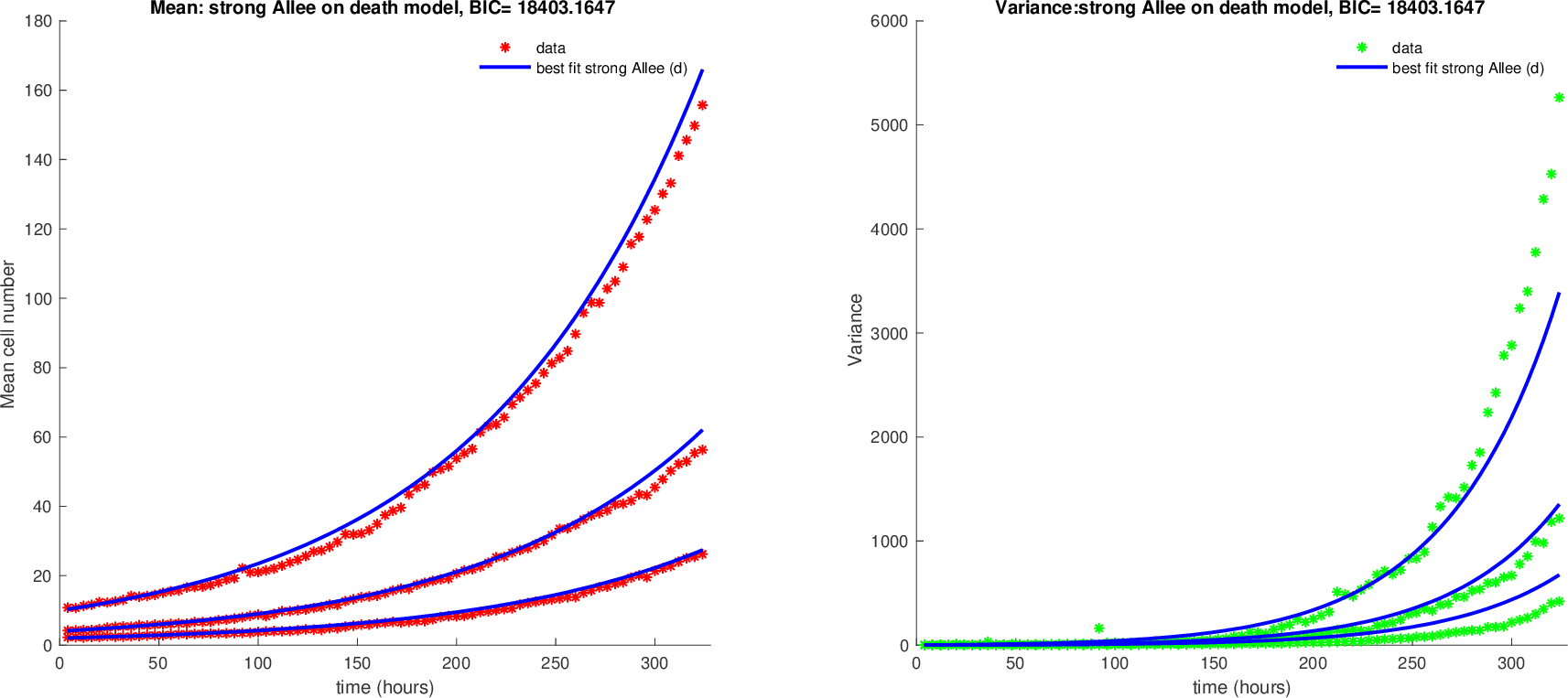

Supplement: S16 Fig — (A) Mean of the data (red) to the best fitting strong Allee on death model mean (blue). (B) Variance of the data (green) to the best fitting strong Allee on death model variance (blue). The data and code used to generate this figure can be found at https://github.com/brocklab/Johnson-AlleeGrowthModel.git. BIC, Bayesian Information Criterion. (TIF) [file pbio.3000399.s018.tif]

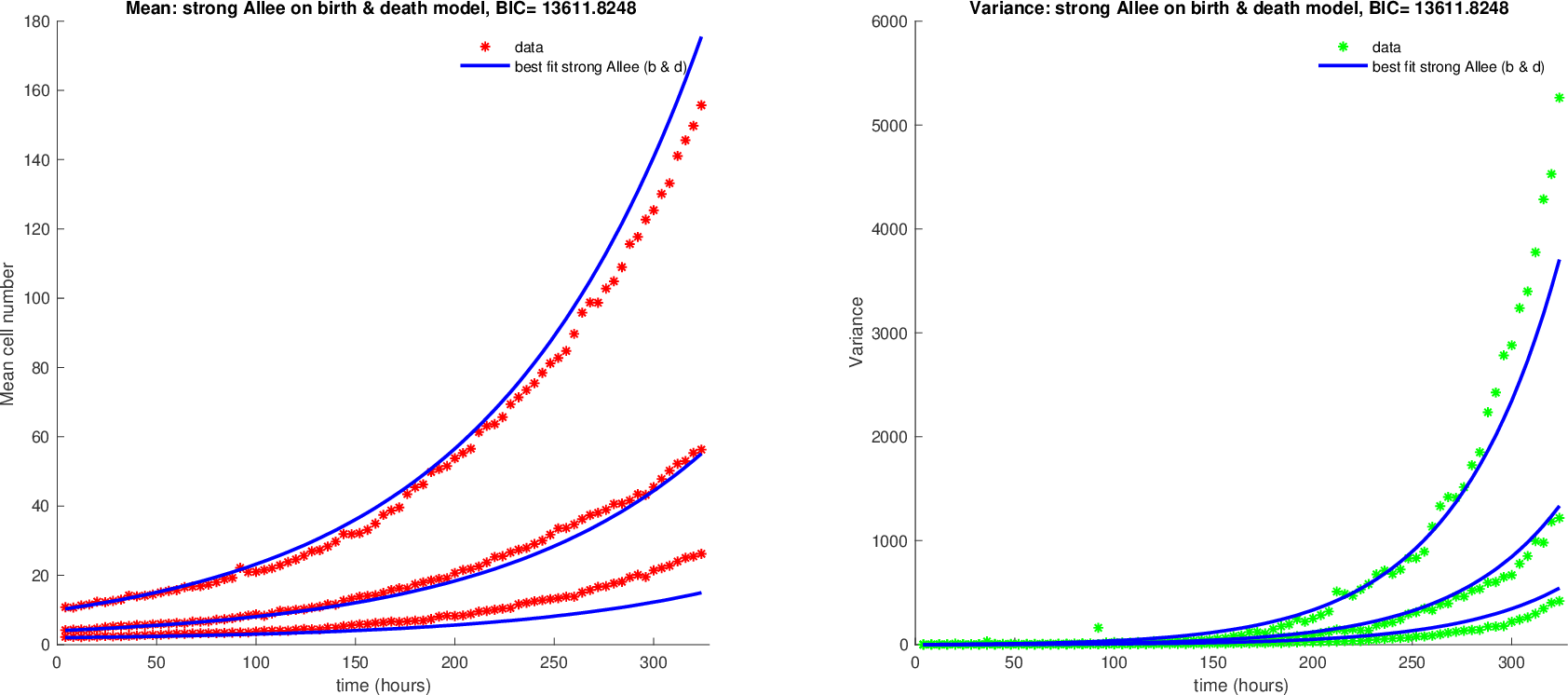

Supplement: S17 Fig — (A) Mean of the data (red) to the best fitting strong Allee on birth and death model mean (blue). (B) Variance of the data (green) to the best fitting strong Allee on birth and death model variance (blue). The data and code used to generate this figure can be found at https://github.com/brocklab/Johnson-AlleeGrowthModel.git. BIC, Bayesian Information Criterion. (TIF) [file pbio.3000399.s019.tif]

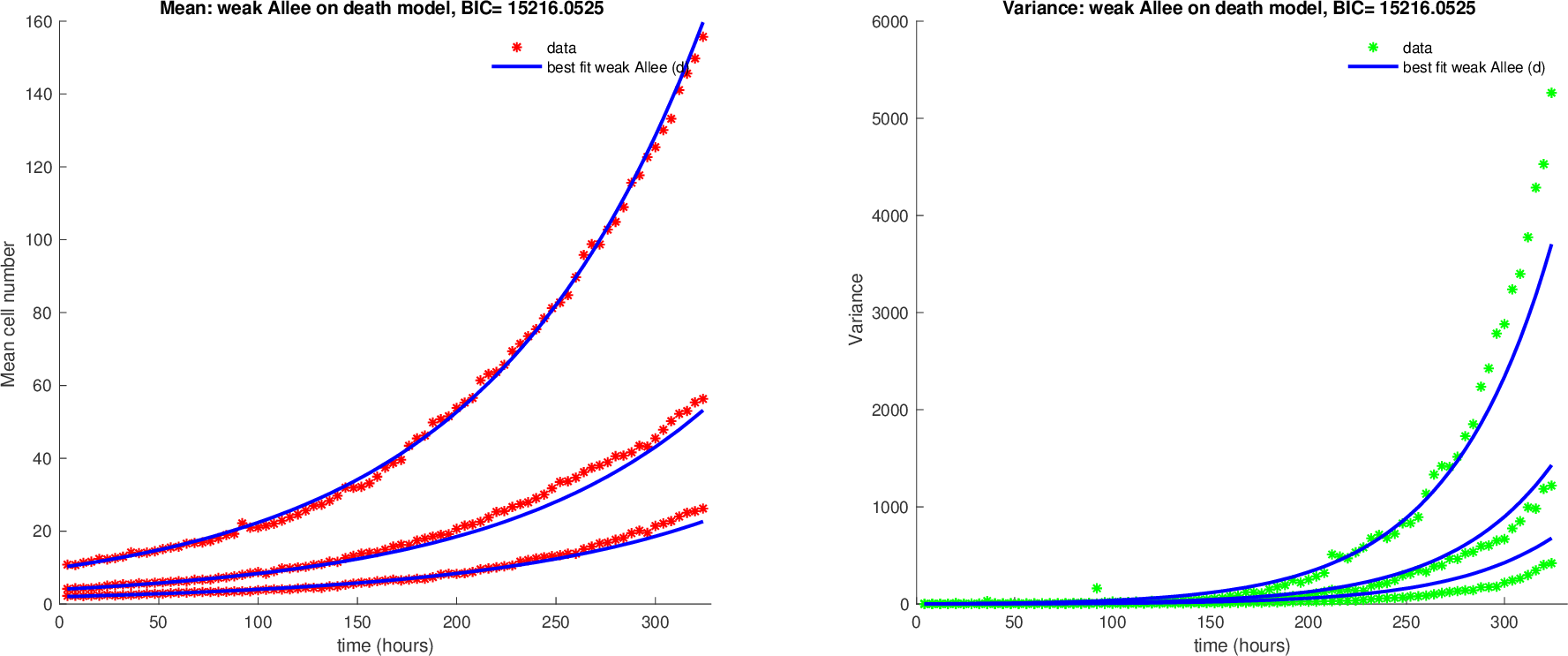

Supplement: S18 Fig — (A) Mean of the data (red) to the best fitting weak Allee on death model mean (blue). (B) Variance of the data (green) to the best fitting weak Allee on death model variance (blue). The data and code used to generate this figure can be found at https://github.com/brocklab/Johnson-AlleeGrowthModel.git. BIC, Bayesian Information Criterion. (TIF) [file pbio.3000399.s020.tif]

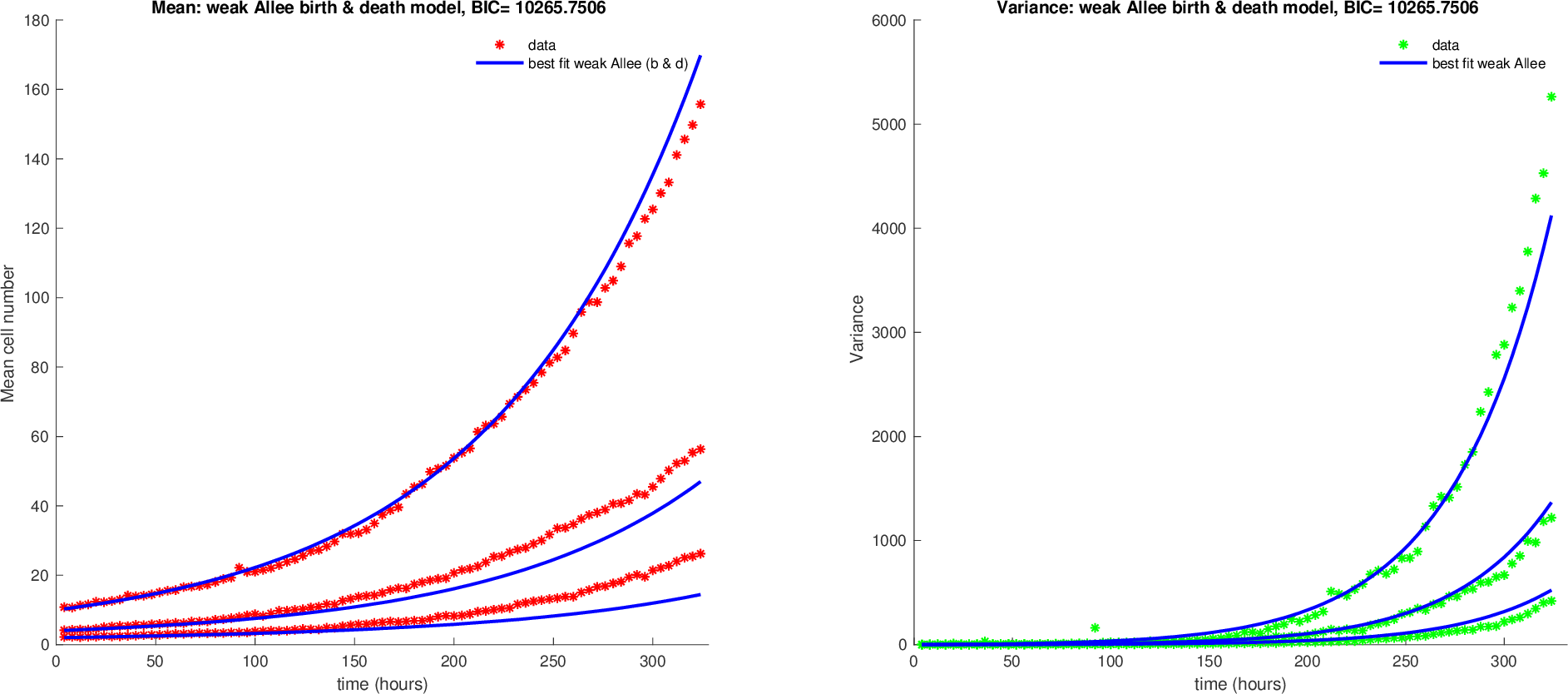

Supplement: S19 Fig — (A) Mean of the data (red) to the best fitting weak Allee on birth and death model mean (blue). (B) Variance of the data (green) to the best fitting weak Allee on birth and death model variance (blue). The data and code used to generate this figure can be found at https://github.com/brocklab/Johnson-AlleeGrowthModel.git. BIC, Bayesian Information Criterion. (TIF) [file pbio.3000399.s021.tif]
