# Supplementary material for: Cancer cell population growth kinetics at low densities deviate from the exponential growth model and suggest an Allee effect
Source: PLoS Biol. 2019 Aug 5;17(8):e3000399. doi: 10.1371/journal.pbio.3000399 (PMC6695196; doi:10.1371/journal.pbio.3000399)
Supplement: S1 Text — (DOCX) [file pbio.3000399.s022.docx]

**S1 Text. Stochastic model simulation using the Gillespie Algorithm**

To evolve the stochastic model forward, we implement the Gillespie algorithm (1,2)

by initializing the number of cells to begin with and the rate parameters that describe the probability of either a birth or death event. The Monte Carlo step is performed to generate random numbers that determine the next event (either a birth or a death) to occur as well as the time interval until that event occurs. The probability of a given event to be chosen is proportional to the reaction propensity and the time interval to the next event is exponentially distributed with mean of the reciprocal of the sum of the probability of any of the events occurring. This process is described below:

$$\begin{matrix} r1=rand\in[0,1] \\ r2=rand\in[0,1] \\ r1<\frac{P(birth)}{\begin{matrix} P(birth)+P(death) \\ N=N+1 \\ else \\ N=N-1 \end{matrix}} \\ \Delta t=\frac{-\log(r2)}{P(birth)+P(death)} \end{matrix}$$

Where P(birth) and P(death) are specific to the stochastic model structure (See S2 Table for definitions that correspond to each model). These steps were repeated for up to 5000 repetitions. Because the time step was probabilistic, each stochastic trajectory was sampled to obtain a uniform time interval where the number of cells was recorded at each time based on the number at the event equal to or before the interval.

**References:**

1. Gillesple DT. Exact Stochastic Simulation of Coupled Chemical Reactions. J Phys Chem. 1977;81(25):2340–61.

2. Gillespie DT. The chemical Langevin equation. J Chem Phys. 2014;297(2000).
