# Supplementary material for: Cancer cell population growth kinetics at low densities deviate from the exponential growth model and suggest an Allee effect
Source: PLoS Biol. 2019 Aug 5;17(8):e3000399. doi: 10.1371/journal.pbio.3000399 (PMC6695196; doi:10.1371/journal.pbio.3000399)
Supplement: S2 Text — (DOCX) [file pbio.3000399.s023.docx]

**S2 Text. Derivation of the moment-closure approximation for the first moment of the birth-death model**

Starting from the CME for the birth-death model below, we apply the ∑n^m^th operator (in the case of the first moment m=1) over the time derivative of the probability of their being n cells at time t to obtain the dime derivative of the expectation of n.


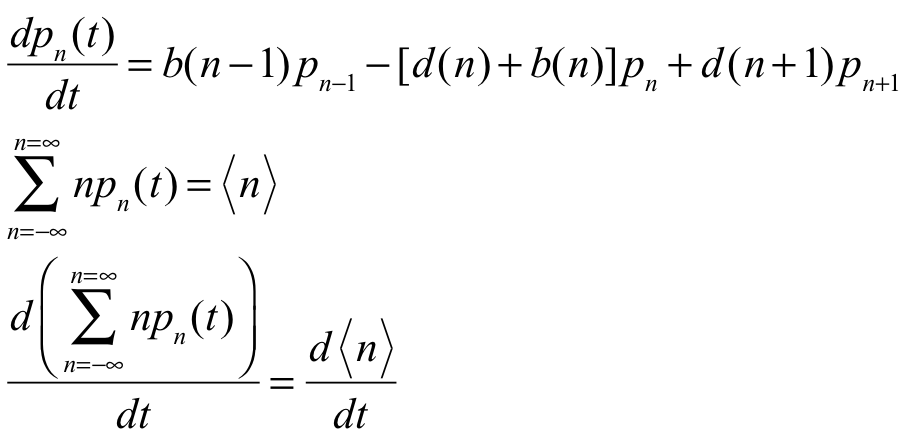


We apply the ∑n^m^th operator to each term on the RHS of the CME. We can then use the fact that the summation from -∞ to ∞ is the same over n-1, n+1, and n to transform the p_n+1_ and p_n-1_ to p_n_s by transforming each term on the RHS so that n-1= n, and substituting into each multiplicative term accordingly as shown below:


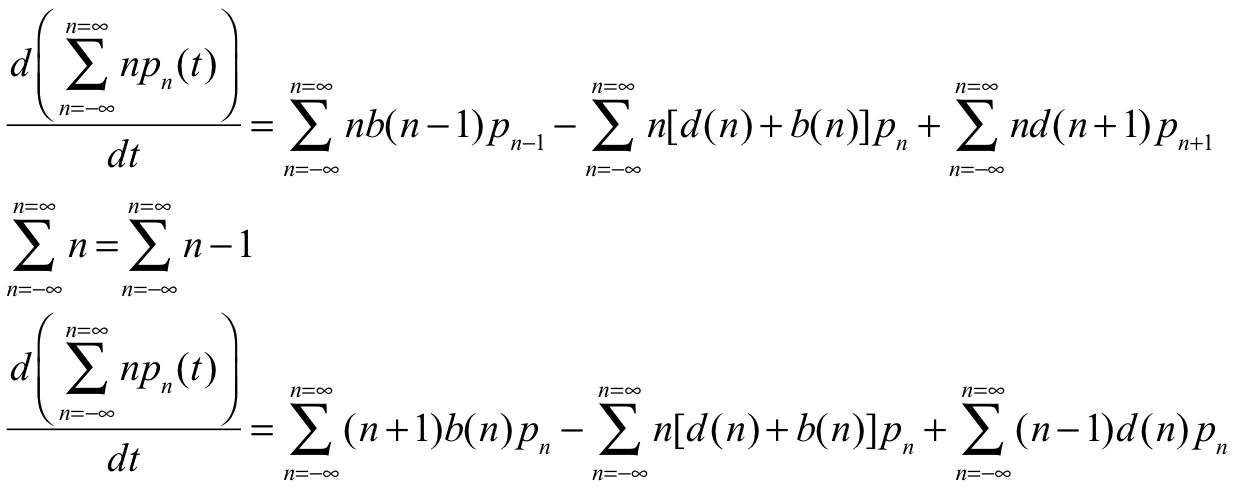


Factoring out pn and applying like terms:


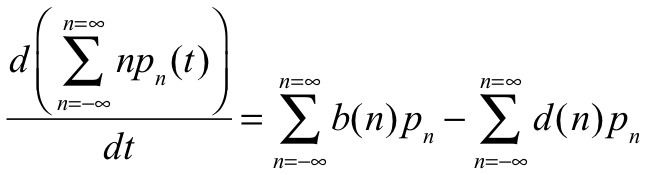


Applying the ∑n^m^th operator gives the time derivative of the expected first moment in terms of the first moment of n itself. In this case, we can solve this analytically to obtain the moment-approach approximation for the mean cell number of the stochastic birth-death process described by exponential growth with a growth rate equal to the birth rate minus the death rate.


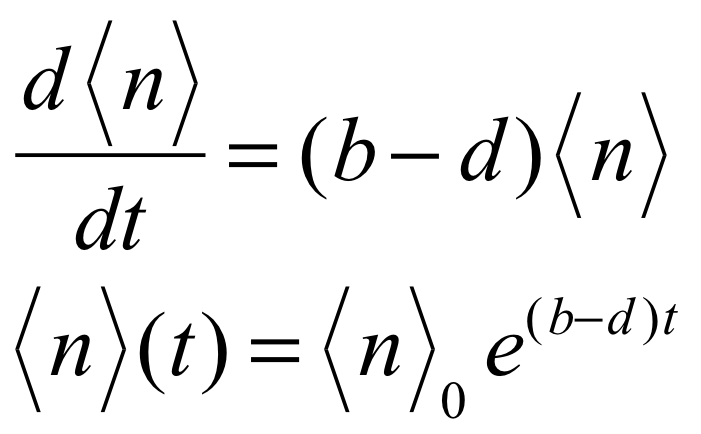


This same derivation(1) was repeated to find up to the 4^th^ moment, and the definition of the variance was used to derive the expected time-derivative of the variance use to identify the magnitude of the birth and death rate.

**References:**

1. Houchmandzadeh B. Extracting moments from Master Equations. ArXiv. 2009;1(2):1–14.
