# Supplementary material for: Cancer cell population growth kinetics at low densities deviate from the exponential growth model and suggest an Allee effect
Source: PLoS Biol. 2019 Aug 5;17(8):e3000399. doi: 10.1371/journal.pbio.3000399 (PMC6695196; doi:10.1371/journal.pbio.3000399)
Supplement: S3 Text — (DOCX) [file pbio.3000399.s024.docx]

**S3 Text. Confirmation that derivations of mean and variance for each model match the mean and variance from simulated data with known parameters**

The moment-approximation derivations from the CME were confirmed to match the measured moments from simulated data from the Gillespie algorithm. In S2 Fig, five thousand trajectories are simulated from the stochastic birth-death model (Eq. 7 & 8) with an initial cell number on N_0_=5 and a birth rate of b = 0.0238 cells/ hour and a death rate of d = 0.005 cells/hour (S2A Fig). The stochastic simulation trajectories were sampled every 4 hours, and the mean and variance in cell number were calculated at each time point (S2B Fig). S2C shows the measured mean from simulated data and the expected mean are a near perfect match, and S2D shows the measured and expected variance are as well. See S3-S8 Figs for confirmation that the expected mean and variance from the remaining six stochastic models match the measured mean and variance from simulated data.
