# Supplementary material for: Cancer cell population growth kinetics at low densities deviate from the exponential growth model and suggest an Allee effect
Source: PLoS Biol. 2019 Aug 5;17(8):e3000399. doi: 10.1371/journal.pbio.3000399 (PMC6695196; doi:10.1371/journal.pbio.3000399)
Supplement: S4 Text — (DOCX) [file pbio.3000399.s025.docx]

**S4 Text. Theoretical identifiability of the structural models using the differential algebra approach applied to the simple birth-death model as an example**

The differential algebra approach(1, 2) requires equations be written to describe the process being modeled and the measurements available. In this case, we are modeling the linear two compartment system of differential equations that describes the time evolution of the mean and the variance in the bulk cell number. Because our experimental system is able to capture a high number of growth trajectories at low initial cell densities, we are able to measure the mean and variance throughout time for each initial condition. For the birth and death model, this leads to the following set of model and measurement equations from Table 1.

$$\begin{matrix} \mathop{n}\limits^{\bullet} =(b-d)n \\ \mathop{V}\limits^{\bullet} =2(b-d)V+(b+d)n \\ y_{1}=n \\ y_{2}=V \end{matrix}$$

Next, we can rewrite these in terms of the measurable outputs y1 and y2. We solve for n from the derivative of V differentiate, then set this equal to the derivative of n.

$$\begin{matrix} y_{1}=\frac{\mathop{y_{2}}\limits^{\bullet} -2(b-d)y_{2}}{b+d} \\ \mathop{y_{1}}\limits^{\bullet} =\frac{\mathop{y_{2}}\limits^{\bullet\bullet} }{b+d}-\frac{2(b-d)\mathop{y_{2}}\limits^{\bullet} }{b+d}=(b-d)y_{1} \\ 0=\frac{\mathop{y_{2}}\limits^{\bullet\bullet} }{b+d}-\frac{2(b-d)\mathop{y_{2}}\limits^{\bullet} }{b+d}-(b-d)y_{1} \end{matrix}$$

The coefficients in front of the measurable outputs are the identifiable parameter combinations, which we set equal to a_1_, a_2_, and a_3_. We then use substitution and replacement to solve for the parameters b & d in terms of the identifiable combinations.

$$\begin{matrix} d=\frac{2-a_{2}}{4} \\ b=\frac{1}{a_{1}}+\frac{a_{2}}{4}-\frac{1}{2} \end{matrix}$$

If we can isolate each parameter in terms of identifiable combinations (a1, a2, and a3) alone, then the parameters are structurally identifiable, as is shown here by the isolation of b & d. Note that the unique identifiability of b and d, not just (b-d) would not be identifiable without the measurement of the variance (specifically because the time derivative of the variance is proportional to b+d), as is explained in Figure S1. We performed this analysis for all seven stochastic model structures, and found all parameters to be uniquely structurally identifiable.

References:

1. Meshkat N, Sullivant S, Eisenberg M. Identifiability Results for Several Classes of Linear Compartment Models. Bull Math Biol. 2015;77(8):1620–51.

2. Brouwer AF, Meza R, Eisenberg MC, Arbor A. A systematic approach to determining the identifiability of multistage carcinogenesis models. Risk Anal. 2018;37(7):1375–87.
