# Supplementary material for: Cancer cell population growth kinetics at low densities deviate from the exponential growth model and suggest an Allee effect
Source: PLoS Biol. 2019 Aug 5;17(8):e3000399. doi: 10.1371/journal.pbio.3000399 (PMC6695196; doi:10.1371/journal.pbio.3000399)
Supplement: S5 Text — BIC, Bayesian Information Criterion. (DOCX) [file pbio.3000399.s026.docx]

**S5 Text. Model Selection using Bayesian Information Criterion and BIC weights**

To investigate competing hypotheses about the underlying structure of tumor growth dynamics, the seven distinct stochastic models were compared using the Bayesian information criterion (BIC) (1, 2). The BIC takes into account both goodness of fit of the model and penalizes for complexity of the model in terms of number of parameters, and has been shown to be an inexpensive approximation to the Bayes factors, which gives the favor of a model over another (2). In order to ensure the method was not overweighing goodness of fit, the data was down-sampled from the true data collection interval of every 4 hours to every 36 hours to demonstrate that down-sampling changed the magnitudes of the BIC values but did not affect the order of the BIC values of each model relative to one another (S11 Fig). To evaluate statistical significance between models with BIC values that were very close to one another, the methods presented in Waenmakers & Farrell et al (3) of BIC weighting were used which are given by:

$$w_{i}(BIC)=\frac{\exp(-\frac{1}{2}\Delta_{i}(BIC))}{\sum_{k=1}^{K} \exp(-\frac{1}{2}\Delta_{k}(BIC))}$$

From this equation, each model is assigned a relative weight, whose sum add to 1 based on the probability that it is the most parsimonious model to describe the data.

References:

1. Raftery A. Bayes Factors and BIC. Sociol Methods Res. 1999;27(3):411–27.

2. Loos C, Moeller K, Fröhlich F, Hucho T, Hasenauer J. A Hierarchical, Data-Driven Approach to Modeling Single-Cell Populations Predicts Latent Causes of Cell-To-Cell Variability. Cell Syst. 2018;6(5):593–603.e13.

3. Wagenmakers E, Farrell S. AIC model selection using Akaike weights. Psychon Bull Rev. 2004;11(1):192–6.
